# Health-Related Quality of Life for Patients Receiving Tumor Treating Fields for Glioblastoma

**DOI:** 10.3389/fonc.2021.772261

**Published:** 2021-12-02

**Authors:** Joshua D. Palmer, Gordon Chavez, Wesley Furnback, Po-Ya Chuang, Bruce Wang, Christina Proescholdt, Chao-Hsiun Tang

**Affiliations:** ^1^ The James Cancer Hospital and Solove Research Institute at the Ohio State University, Columbus, OH, United States; ^2^ Department of Global Value, Novocure, New York, NY, United States; ^3^ Department of Value and Access, Real Chemistry, New York, NY, United States; ^4^ Department of Global Value, Novocure, Root, Switzerland; ^5^ School of Health Care Administration, Taipei Medical University, Taipei, Taiwan

**Keywords:** glioblastoma, tumor treating fields, quality of life, EQ-5D, real-world evidence

## Abstract

**Background:**

To date, there has been no large-scale, real-world study of the health-related quality of life outcomes for patients using tumor treating fields (TTFields) therapy for glioblastoma (GBM) treatment.

**Methods:**

A survey was mailed to 2,815 patients actively using TTFields for treatment of GBM in the USA (*n* = 2,182) and Europe (*n* = 633). The survey included patient-reported demographic and clinical information, as well as EuroQol’s EQ-5D-5L and visual analogue scale (EQ-VAS) overall health score.

**Results:**

A total of 1,106 applicable patients responded to the survey (USA = 782 and Europe = 324), with a mean age of 58.6 years (SD = 12.3). The average time since diagnosis and time using TTFields were 21.5 months (SD = 25.1) and 13.5 months (SD = 13.2), respectively. Over 61% of patients had been diagnosed at least 1 year prior and 28.4% at least 2 years prior; 45 patients (4.2%) had been diagnosed at least 5 years prior. Progressed disease was reported in 307 patients, while 690 reported non-progressed disease. Regression analyses showed that GBM disease progression and older age had predictable negative associations (*p* < 0.001) with most EQ-5D-5L dimensions and the EQ-VAS. However, longer time since diagnosis was associated with improved self-care (*p* < 0.05), usual activities (*p* < 0.01), and EQ-VAS (*p* < 0.05) overall and in patients with progressed disease (*p* < 0.01, *p* < 0.05, and *p* < 0.01, respectively). Additionally, longer time using TTFields was associated with improved mobility (*p* < 0.05), self-care (*p* < 0.001), usual activities (*p* < 0.01), and EQ-VAS (*p* < 0.01) overall; with improved EQ-VAS in progression-free patients (*p* < 0.05); and with improved mobility (*p* < 0.05), self-care (*p* < 0.01), usual activities (*p* < 0.05), and EQ-VAS (*p* < 0.05) in patients with progressed disease.

**Conclusion:**

This is the largest real-world study of patient-reported quality of life in GBM and TTFields treatment to date. It shows unsurprising negative associations between quality of life and disease progression and older age, as well as more novel, positive associations between quality of life and longer time since diagnosis and time using TTFields therapy.

## Introduction

Glioblastoma (GBM) is the most common and aggressive primary brain malignancy, and the incidence rates across the world have been increasing (1–3). Patients with GBM experience symptoms at diagnosis, such as fatigue, seizures, cognitive effects, and headaches, which often worsen over time due to the aggressive nature of GBM (4–6). GBM is characterized by high rates of initial mortality with a 2-year survival rate of 18% (7) and median overall survival of 14.6 months (8). However, 2-year conditional survival rates have been shown to improve from diagnosis (10%) through 4 years post-diagnosis (67%) (9).

There have only been two approved therapeutic options for patients with newly diagnosed GBM. In 2005, temozolomide, an oral maintenance chemotherapy, was approved through a landmark study reporting a significant increase in overall survival for GBM patients treated with temozolomide + radiotherapy compared to radiotherapy alone (8). In 2015, tumor treating fields (TTFields), a device emitting low-intensity alternating electric fields at intermediate frequencies, was approved for newly diagnosed GBM after demonstrating a significant increase in median overall survival (10).

Despite only two approved therapeutic options in newly diagnosed GBM, survival has been increasing over time (3, 7). A previously developed integrated survival model for TTFields + maintenance temozolomide estimated that over 20% of patients surviving 2 years will survive through 10 years (11). While the number of long-term survivors (patients surviving longer than the median of 15 months since diagnosis) continues to grow, there has been little research into the health-related quality of life (HRQoL) of these patients. A review of the literature assessing HRQoL within long-term survivors of GBM returned two studies, both of which involved a small number of patients and neither of which included EQ-5D (12, 13).

Studies evaluating HRQoL in GBM patients using TTFields have mostly been in the clinical trial setting, and real-world analyses have not included long-term survivors. HRQoL was included in the EF-14 trial and compared the TTFields + temozolomide and temozolomide-alone arms, which yielded no significant differences through 12 months post-diagnosis (14, 15). However, there are no available data for patients on TTFields evaluating differences in HRQoL between patients with progressed disease and progression-free patients or according to time since diagnosis beyond 12 months or time using TTFields therapy. Because the number of long-term GBM survivors is increasing, there is a need to understand the health-related quality of life these patients experience, which will allow stakeholders to better estimate the benefits of effective therapies. The objective of this study was to conduct a large-scale, real-world, cross-sectional HRQoL survey of patients currently receiving treatment with TTFields for GBM in the USA and Europe.

## Methods

### Study Participants

All patients being treated with TTFields for GBM outside of a clinical trial with a primary address in the USA or Europe (Germany, Austria, and Switzerland) were eligible for inclusion in this study. This study was evaluated by the WIRB Copernicus Group (WCG) IRB and granted an exemption because the research only involved survey procedures and the information obtained was recorded such that the identity of the human subjects could not be ascertained, directly or through identifiers linked to the subjects.

### Survey Design and Administration

The cover letter invited patients to participate in the survey and noted that participation was voluntary. Patients were informed that their responses would be completely anonymized and that their participation and responses would in no way affect their care or coverage.

**Figure 1:**
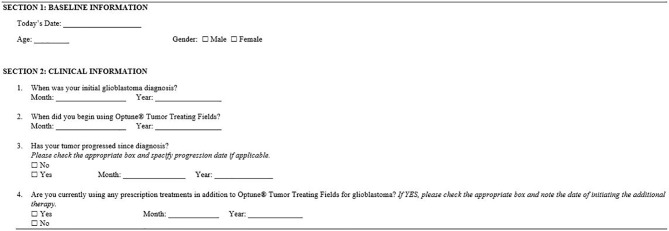
Survey of baseline demographic and clinical information. All items included in the survey were self-reported by respondents. Respondents also received their country-specific EuroQol’s EQ-5D-5L questionnaire and EuroQol’s visual analogue scale (EQ-VAS).

The patient survey (**Figure 1**) captured demographic and clinical information and included EuroQol’s EQ-5D-5L questionnaire and EuroQol’s visual analogue scale (EQ-VAS) (16). Country-specific EQ-5D questionnaires were used and other survey materials were professionally translated into the respondents’ local language.

Within the few real-world studies assessing HRQoL in GBM patients, a variety of measurements have been used, with the European Organization for Research and Treatment of Cancer Quality of Life Questionnaire (EORTC QLQ-C30) being the most frequent (12, 17–20). The EQ-5D questionnaire has been utilized in some instances, but has never been used to evaluate patients on TTFields exclusively (17, 21–23). This study selected the EQ-5D questionnaire due to its broad applicability and validation across geographic areas, the relatively low survey burden, and ability for it to be self-administered.

### Statistical Analysis

Univariate and multivariate linear regression analyses were conducted with the survey results to understand the impact of demographics, treatment, and clinical characteristics on patients’ HRQoL. These analyses consisted of regressing patient-reported EQ-5D dimensions and EQ-VAS on the patient demographics and clinical variables, including age, gender, current treatment (TTFields alone or TTFields + other therapy), disease progression status (non-progressed or progressed), time since diagnosis, and time on TTFields.

Because of the discrete, ordinal nature of the five dimensions of the EQ-5D-5L, the linear regression analyses presented here were supplemented with univariate and multivariate *ordinal regression* analyses.

Time since diagnosis and time on TTFields were also studied in their logarithmic form; additionally, a binary time from diagnosis variable was analyzed using a threshold of 15 months, as this is recognized as the current median overall survival for patients with GBM (8). For the purpose of this study, patients surviving past the historical median overall survival of 15 months in GBM are considered “long-term survivors.” Time since diagnosis and time on TTFields were highly correlated and hence treated separately in the regression analysis to prevent collinearity problems. Subgroup regression analyses were also performed separately on patients who had experienced progression of their GBM disease (progressed) and those who had not (non-progressed).

Since multiple comparisons are being made, *p*-values were adjusted using the Bonferroni method, which is known to be the most conservative with regard to minimizing type I errors. In the univariate regression results for the overall sample (**Table 3**), the raw *p*-values were multiplied by 6, as this was the number of variables we compare (age, gender, progression status, other therapies, time since diagnosis, and time on TTFields). For the multivariate results in the overall sample (**Tables 4** and **5**), the raw *p*-values were multiplied by 2, as time since diagnosis and time on TTFields are being compared. In the subgroup univariate analysis of patients with non-progressed *vs.* progressed disease (**Table 6**), the raw *p*-values were multiplied by 5 (the above-mentioned factors minus progression status) and by 2 in the comparison of time since diagnosis and time on TTFields analysis (**Tables 7** and **8**). An adjusted *p*-value of less than 0.05 was considered statistically significant.

The completeness of the data was at least 90% in all items. Patients were excluded from any analyses that included variables directly or indirectly linked to missing or implausible responses (**Table 1**).

**Table 1:** Data cleaning criteria, number of respondents with unclear results, and action taken.

Variable	Criteria	Respondents	Result
Age	Respondents in the USA aged <22 years	*n* = 3	Removed from sample
Progression status	Respondents without a response to progression (yes/no), but a response for date of progression	*n* = 13	Progression marked “unknown”
	Respondents with a “yes” response to progression, but no response for date of progression	*n* = 34	Progression marked “yes”
	Respondents with a “no” response to progression, but with response for date of progression	*n* = 42	Progression marked “unknown”
Other therapy	Respondents without a response to other GBM therapy (yes/no), but a response for date of initiation	*n* = 6	Other therapy marked “unknown”
	Respondents with a “yes” response to other GBM therapy, but no response for date of initiation	*n* = 79	Other therapy marked “yes”
	Respondents with a “no” response to other GBM therapy, but with response for date of initiation	*n* = 7	Other therapy marked “unknown”
Time since diagnosis	Respondents with a negative value for time since diagnosis	*n* = 1	Time since diagnosis marked “unknown”
	Respondents with diagnosis year prior to date of birth	*n* = 1	Time since diagnosis marked “unknown”
Time on TTFields	Patients with >9 years of time on TTFields	*n* = 5	Time on TTFields marked “unknown”
	Patients with time on TTFields greater than or equal to time since diagnosis	*n* = 26	Time on TTFields marked “unknown”

TTFields, tumor treating fields.

## Results

### Response Rate, Demographics, and Clinical Characteristics

Surveys were mailed to 2,815 patients actively using TTFields for GBM treatment in the USA (2,182 patients) and Europe (633 patients). On thousand one hundred and nine patients responded to the survey, giving an overall response rate of 39.4% (USA = 35.8% and Europe = 51.2%). There were 1,106 patients included in the final analysis after three US-based patients were removed because they were under 22 years of age and hence off the Food and Drug Administration (FDA)-approved label for use of TTFields.

The sample was mostly males (*n* = 651, 62.24%), with a mean patient age of 58.6 years (SD = 12.3) and median age of 61 years (min = 21, max = 86).

Most of the sample (*n* = 690, 62.4%) had not experienced disease progression as of the survey date (27.8% with progressed disease and 9.9% had unknown progression status). Within patients reporting progressed disease (*n* = 307), the mean (SD) and median (IQR) time since progression at the time of the survey were 6.8 (10.7) and 3 (1–8) months, respectively. Time since progression was not known for 35 patients reporting progressed disease. Of the 307 patients who reported progressed disease, 54 (17.6%) reported that their disease had progressed prior to starting TTFields therapy. Within these patients, the mean (SD) and median (IQR) time between progression and TTFields start were 5.8 (12.6) and 2 (1–4) months, respectively. Two hundred thirteen patients (69.4%) reported that their disease had progressed after starting TTFields therapy, with a mean (SD) and median (IQR) time between TTFields start and progression of 10 (13.2) and 6 (3–11) months, respectively. Forty patients with progressed disease (13%) did not report sufficient information to evaluate the time of progression relative to TTFields start.

The mean time since diagnosis was 21.54 months (SD = 25.1), and the median time since diagnosis was 14 months (IQR = 9–26). Time since diagnosis was not known for 22 (2.0%) patients. Over 61% of patients who reported their diagnosis date had been diagnosed at least 1 year prior to taking the survey, and 28.4% of patients had been diagnosed at least 2 years prior. There were 45 patients (4.2%) with a time since diagnosis of five or more years.

The mean time on TTFields was 13.51 months (SD = 13.2), and the median time on TTFields was 9 months (IQR = 4–19). Over half (*n* = 613, 60.3%) of patients had been using TTFields for less than 1 year at the time of the survey. There were 90 patients (8.1%) with an unknown time on TTFields. At the time of the survey, 431 patients (39.0%) reported that no other therapies were being used to treat their GBM outside of TTFields. There were 640 patients (57.9%) reporting that other therapies were being used to treat their GBM; the status of the remaining 35 patients (3.2%) was unknown.

### Summary of EQ-5D Results

**Figure 2:**
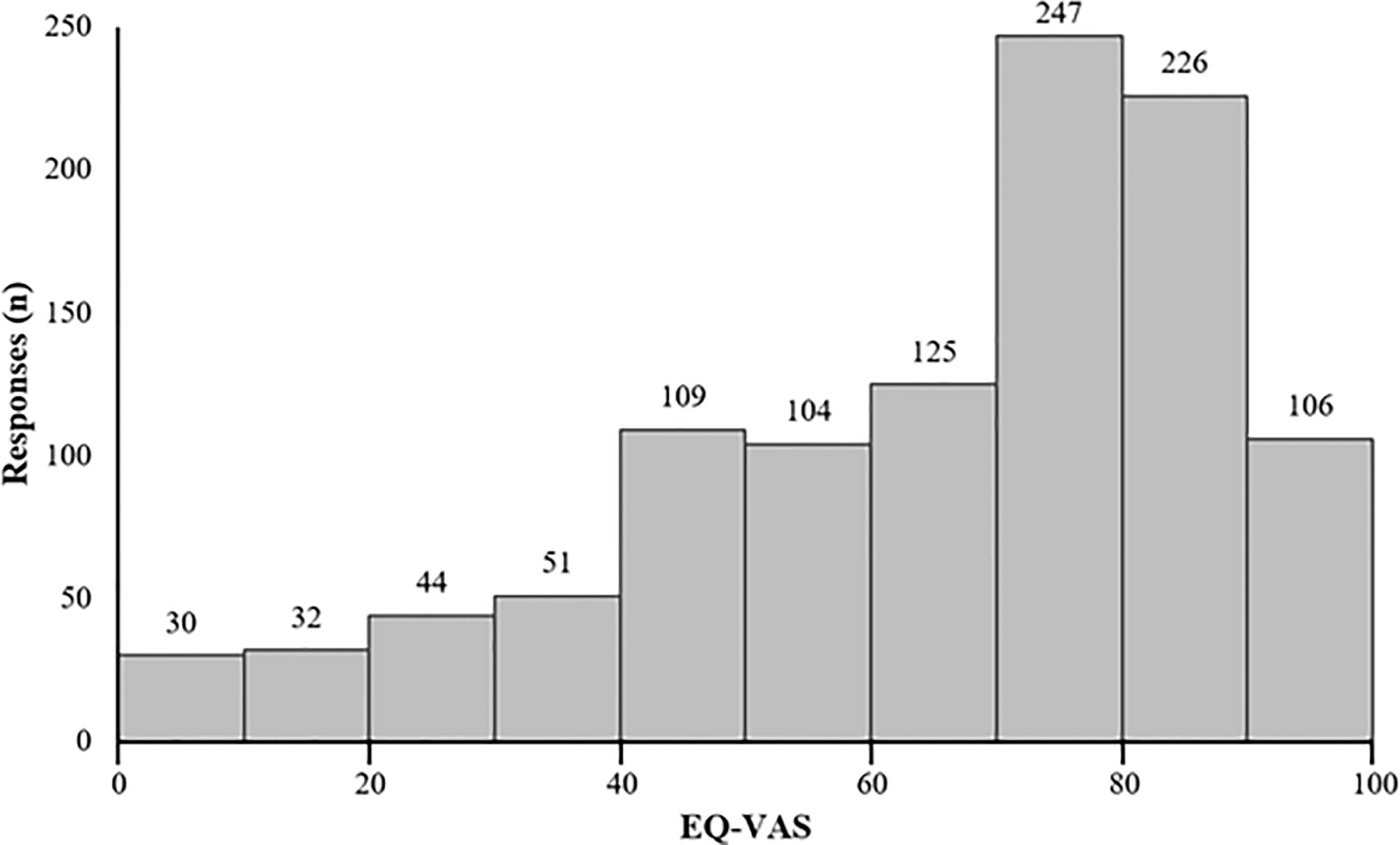
Distribution of patient-reported EuroQol’s visual analogue scale (EQ-VAS) (*n* = 1,074). Higher EQ-VAS values indicated improved self-rated health, and lower values indicated worse self-rated health.

**Figure 3:**
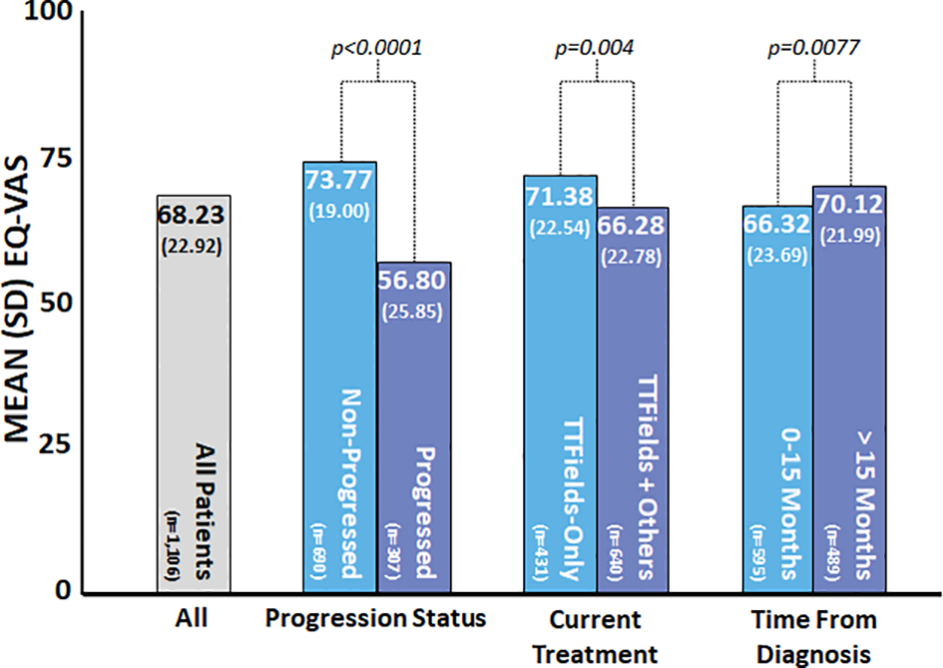
Patient-reported EuroQol’s visual analogue scale (EQ-VAS) by subgroup. EQ-VAS was measured at the time of the survey, and patient and clinical characteristics were self-reported by patients at the time of the survey. Higher values indicated improved self-rated health, and lower values indicated worse self-rated health. *P*-values were calculated using the *t*-test.

The mean reported EQ-VAS score was 68.2 (SD = 22.9), while the median score was 75 (IQR = 55–85) (**Table 2**). VAS scores were clustered toward the high end of the scale (**Figure 2**). Average EQ-VAS scores were significantly higher for patients with non-progressed *vs.* progressed disease (73.77 *vs.* 56.80, *p* < 0.0001), TTFields-only *vs.* TTFields + other treatments (71.38 *vs.* 66.28, *p* = 0.0004), and >15 months since diagnosis *vs.* 0–15 months since diagnosis (70.12 *vs.* 66.32, *p* = 0.0077) (**Figure 3**).

**Table 2:** Overall distribution of levels in the EQ-5D-5L dimensions.

Problems/dimensions	*N*	Mobility	Self-care	Usual activities	Pain/discomfort	Anxiety/depression	EQ-VAS
All patients							
Mean (SD)	1,106	1.95 (1.16)	1.71 (1.15)	2.39 (1.25)	1.70 (0.83)	1.80 (0.85)	68.23 (22.92)
Distribution							
None (1)		48.8%	63.9%	29.3%	50.2%	42.0%	–
Slight (2)		24.3%	16.6%	30.3%	33.2%	40.9%	–
Moderate (3)		15.1%	10.1%	22.3%	13.7%	12.9%	–
Severe (4)		7.1%	3.7%	9.1%	2.6%	3.3%	–
Extreme (5)		4.7%	5.7%	9.1%	0.4%	0.9%	–
Progression status, mean (SD)							
Non-progressed	690	1.71 (0.98)	1.46 (0.89)	2.15 (1.13)	1.6 (0.76)	1.7 (0.81)	73.77 (19)
Progressed	307	2.37 (1.35)	2.15 (1.4)	2.85 (1.33)	1.89 (0.94)	2.01 (0.9)	56.8 (25.85)
Current treatments, mean (SD)							
TTFields only	431	1.84 (1.16)	1.59 (1.09)	2.23 (1.26)	1.61 (0.8)	1.73 (0.81)	71.38 (22.54)
TTFields + others	640	2.01 (1.16)	1.78 (1.17)	2.47 (1.22)	1.76 (0.84)	1.84 (0.86)	66.28 (22.78)
Time from diagnosis, mean (SD)							
0–15 months	595	1.99 (1.2)	1.79 (1.21)	2.51 (1.29)	1.71 (0.83)	1.85 (0.89)	66.32 (23.69)
>15 months	489	1.9 (1.12)	1.61 (1.08)	2.25 (1.18)	1.69 (0.83)	1.76 (0.8)	70.12 (21.99)

Lower scores indicate less impairment and higher scores more impairment in health-related quality of life (HRQoL) for the EQ-5D subscales of mobility, self-care, usual activities, pain/discomfort, and anxiety/depression. For EQ-VAS, higher values indicate improved self-rated health and lower values indicate worse self-rated health.

EQ-VAS, EuroQol’s visual analogue scale; TTFields, tumor treating fields.

Within the overall sample, the highest percentage of respondents reported no problems in the EQ-5D-5L dimensions of mobility (48.8%), self-care (63.9%), pain/discomfort (50.2%), and anxiety/depression (42.0%) (**Table 2**). Within usual activities, 29.3% of patients reported no problems compared to 30.3% of patients reporting slight problems.

There were 164 patients (15.8%) who reported no problems in any of the EQ-5D-5L dimensions of mobility, self-care, usual activities, pain/discomfort, or anxiety/depression, indicating very high HRQoL along these dimensions. Additionally, over 51% of patients reported only no problems or slight problems in every dimension, and 79% reported at worst moderate problems in any dimension. Less than 10% of the sample reported extreme problems in any of the five measured EQ-5D-5L dimensions. Seventy patients did not provide complete EQ-5D-5L survey responses.

### Regression Analyses

The univariate analysis showed both positive associations/improvements (dark shaded values) and negative associations/declines (light shaded values) in HRQoL, with variations in the demographic and clinical variables (**Table 3**). We summarize these results in this subsection.

**Table 3:** Univariate regression results.

Univariate Analysis	Mobility			Self-care			Usual activities			Pain/discomfort			Anxiety/depression			EQ-VAS		
	Effect	SE	*p*-value	Effect	SE	*p*-value	Effect	SE	*p*-value	Effect	SE	*p*-value	Effect	SE	*p*-value	Effect	SE	*p*-value
Gender																		
Female (ref.)																		
Male	−0.076	0.075	1.8342	0.011	0.073	5.3184	0.094	0.081	1.4568	−0.042	0.053	2.5836	−0.070	0.055	1.2462	−1.339	1.465	2.1642
Age	0.021	0.003	<0.001	0.013	0.003	<0.001	0.015	0.003	<0.001	−0.003	0.002	1.3872	−0.001	0.002	3.7896	−0.202	0.057	0.0024
Progression status																		
Non-progressed																		
Progressed	0.656	0.077	<0.001	0.692	0.074	<0.001	0.696	0.083	<0.001	0.289	0.057	<0.001	0.311	0.058	<0.001	−16.973	1.483	<0.001
Current treatment																		
TTFields only																		
TTFields + others	0.168	0.073	0.1272	0.189	0.071	0.048	0.242	0.078	0.0114	0.152	0.052	0.0198	0.104	0.053	0.2886	−5.106	1.433	0.0024
Time since diagnosis																		
0–15 months																		
>15 months	−0.090	0.072	1.2486	−0.182	0.071	0.06	−0.259	0.077	0.0042	−0.017	0.051	4.4196	−0.087	0.053	0.5838	3.800	1.421	0.045
Time since diagnosis	−0.001	0.001	3.6018	−0.002	0.001	1.2486	−0.003	0.002	0.3354	0.000	0.001	4.2576	−0.001	0.001	3.6606	0.066	0.028	0.1134
Log(Time since diagnosis)	−0.044	0.048	2.1426	−0.099	0.047	0.2148	−0.139	0.051	0.0396	0.008	0.034	4.917	−0.029	0.035	2.4438	2.286	0.950	0.0966
Time on TTFields	−0.008	0.003	0.0276	−0.012	0.003	<0.001	−0.013	0.003	<0.001	−0.004	0.002	0.3342	−0.003	0.002	0.7782	0.219	0.055	<0.001
Log(Time on TTFields)	−0.121	0.037	0.0066	−0.165	0.036	<0.001	−0.172	0.039	<0.001	−0.051	0.026	0.3132	−0.047	0.027	0.5034	2.631	0.731	0.0018

Entries with light shading denote statistically significant deterioration, while those with dark shading indicate statistically significant improvement. Within the EQ-5D subscales (mobility, self-care, usual activities, pain/discomfort, and anxiety/depression), lower scores indicate less impairment and higher scores more impairment in health-related quality of life (HRQoL). For EQ-VAS, higher values indicate improved self-rated health and lower values indicate worse self-rated health. Quoted p-values = raw p-values × 6 to adjust for multiple comparisons.

EQ-VAS, EuroQol’s visual analogue scale; TTFields, tumor treating fields.

Older age was significantly correlated with worse patient-reported outcomes on mobility (*p* < 0.001), self-care (*p* < 0.001), and usual activities (*p* < 0.001), along with EQ-VAS (*p* = 0.0024). Disease progression had a large and significant negative impact on patients’ HRQoL across all reported dimensions and EQ-VAS (*p* < 0.001 for all). Using other treatments for GBM in addition to TTFields was also correlated with more problems in self-care (*p* = 0.048), usual activities (*p* = 0.0114), pain/discomfort (*p* = 0.0198), and EQ-VAS (*p* = 0.0024).

However, the univariate analysis also showed a significant correlation between longer time since diagnosis and improved patient-reported outcomes. Patients who had been diagnosed at least 15 months prior to the survey reported significantly improved usual activities (*p* = 0.0042) and EQ-VAS (*p* = 0.045) compared to patients with less than 15 months since diagnosis.

Highly significant, positive associations with mobility, self-care, usual activities, and EQ-VAS were found for longer time on TTFields (*p* = 0.0066, *p* < 0.001, *p* < 0.001, *p* < 0.001, respectively). These observations were further investigated using multivariate regression analysis.

The negative associations from age and disease progression were observed in all multivariate analyses. The positive associations with longer time since diagnosis were observed for self-care (*p* = 0.0296), usual activities (*p* = 0.0076), and EQ-VAS (*p* = 0.0204) in the multivariate setting by regressing on the 15-month survival binary indicator (**Table 4**).

**Table 4:** Multivariate regression results with time since diagnosis.

Multivariate regression	Mobility			Self-care			Usual activities			Pain/discomfort			Anxiety/depression			VAS		
	Effect	SE	*p*-value	Effect	SE	*p*-value	Effect	SE	*p*-value	Effect	SE	*p*-value	Effect	SE	*p*-value	Effect	SE	*p*-value
Gender																		
Female (ref.)																		
Male	−0.024	0.076	1.5126	0.035	0.075	1.273	0.152	0.082	0.1298	−0.046	0.057	0.8408	−0.060	0.058	0.5974	−1.288	1.467	0.7598
Age	0.021	0.003	<0.001	0.013	0.003	<0.001	0.016	0.003	<0.001	−0.002	0.002	0.897	−0.001	0.002	1.463	−0.182	0.058	0.0032
Progression status																		
Non-progressed																		
Progressed	0.688	0.081	<0.001	0.714	0.080	<0.001	0.727	0.088	<0.001	0.291	0.061	<0.001	0.324	0.062	<0.001	−16.922	1.571	<0.001
Current treatment																		
TTFields only																		
TTFields + others	−0.005	0.081	1.8966	−0.015	0.080	1.7024	0.013	0.088	1.7706	0.135	0.061	0.0516	0.000	0.062	1.9968	−0.349	1.568	1.6478
Time since diagnosis																		
0–15 months																		
>15 months	−0.056	0.080	0.9598	−0.191	0.078	0.0296	−0.250	0.086	0.0076	0.043	0.059	0.9382	−0.083	0.061	0.3518	3.948	1.538	0.0204

Entries with light shading denote statistically significant deterioration, while those with dark shading indicate statistically significant improvement. Within the EQ-5D subscales (mobility, self-care, usual activities, pain/discomfort, and anxiety/depression), lower scores indicate less impairment and higher scores more impairment in health-related quality of life (HRQoL). For EQ-VAS, higher values indicate improved self-rated health and lower values indicate worse self-rated health. Quoted p-values = raw p-vales × 2 to adjust for multiple comparisons.

VAS, EuroQol’s visual analogue scale; TTFields, tumor treating fields.

The significant positive association with time on TTFields was maintained for mobility (*p* = 0.0476), self-care (*p* = 0.0006), usual activities (*p* = 0.0022), and EQ-VAS (*p* = 0.0022) in the multivariate analysis (**Table 5**).

**Table 5:** Multivariate regression results with time on TTFields.

Multivariate regression	Mobility			Self-care			Usual activities			Pain/discomfort			Anxiety/depression			VAS		
	Effect	SE	*p*-value	Effect	SE	*p*-value	Effect	SE	*p*-value	Effect	SE	*p*-value	Effect	SE	*p*-value	Effect	SE	*p*-value
Gender																		
Female (ref.)																		
Male	−0.020	0.078	1.5944	0.064	0.076	0.8024	0.133	0.084	0.2266	−0.046	0.059	0.8608	−0.053	0.060	0.752	−0.929	1.506	1.0744
Age	0.021	0.003	<0.001	0.012	0.003	<0.001	0.017	0.003	<0.001	−0.0005	0.002	1.688	0.000	0.002	1.787	−0.186	0.059	0.0032
Progression status																		
Non-progressed																		
Progressed	0.697	0.083	<0.001	0.707	0.081	<0.001	0.724	0.089	<0.001	0.337	0.062	<0.001	0.342	0.063	<0.001	−17.122	1.592	<0.001
Current treatment																		
TTFields only																		
TTFields + others	−0.032	0.083	1.406	−0.047	0.081	1.1276	0.013	0.089	1.7774	0.098	0.063	0.2326	0.005	0.064	1.8684	0.269	1.599	1.7328
Time on TTFields	−0.006	0.003	0.1236	−0.011	0.003	0.0006	−0.011	0.003	0.0022	−0.002	0.002	0.7402	−0.002	0.002	0.5962	0.189	0.058	0.0022

Gender																		
Female (ref.)																		
Male	−0.025	0.078	1.493	0.058	0.077	0.903	0.126	0.085	0.2702	−0.052	0.059	0.7516	−0.054	0.060	0.7412	−0.812	1.511	1.1818
Age	0.022	0.003	<0.001	0.013	0.003	<0.001	0.017	0.003	<0.001	−0.0002	0.002	1.835	0.0000	0.002	1.975	−0.198	0.059	0.0014
Progression status																		
Non-progressed																		
Progressed	0.687	0.083	<0.001	0.696	0.081	<0.001	0.711	0.089	<0.001	0.329	0.063	<0.001	0.338	0.064	<0.001	−16.897	1.598	<0.001
Current treatment																		
TTFields only																		
TTFields + others	−0.053	0.085	1.0708	−0.059	0.084	0.955	−0.001	0.092	1.9904	0.102	0.064	0.2252	−0.010	0.066	1.7602	0.200	1.640	1.8056
Log(Time on TTFields)	−0.095	0.042	0.0476	−0.142	0.041	0.0012	−0.151	0.045	0.0018	−0.024	0.032	0.8858	−0.048	0.032	0.277	2.233	0.812	0.012

Entries with light shading denote statistically significant deterioration, while those with dark shading indicate statistically significant improvement. Within the EQ-5D subscales (mobility, self-care, usual activities, pain/discomfort, and anxiety/depression), lower scores indicate less impairment and higher scores more impairment in health-related quality of life (HRQoL). For EQ-VAS, higher values indicate improved self-rated health and lower values indicate worse self-rated health. Quoted p-values = raw p-values × 2 to adjust for multiple comparisons.

VAS, visual analogue scale; TTFields, tumor treating fields.

### Subgroup Analysis: Progressed *vs.* Non-Progressed Disease

In the univariate analysis (**Table 6**), age had a similar negative association with mobility, self-care, usual activities, and EQ-VAS in both the non-progressed and progressed subgroups. A mild negative association between EQ-VAS (*p* = 0.0245) and the use of TTFields + other treatments compared to TTFields alone was observed in the non-progressed subgroup. This was not observed in the progressed subgroup.

**Table 6:** Univariate regression results for the non-progressed and progressed subgroups.

Univariate regression	Mobility			Self-care			Usual activities			Pain/discomfort				Anxiety/depression				VAS			
	Effect	SE	*p*-value	Effect	SE	*p*-value	Effect	SE	*p*-value	Effect	SE	*p*-value	Effect		SE	*p*-value	Effect		SE	*p*-value
***Non-progressed***																				
Gender																				
Female (ref.)																				
Male	−0.086	0.080	1.4015	−0.019	0.072	3.987	0.114	0.092	1.0855	−0.020	0.061	3.7475	−0.042		0.066	2.6105	−0.853		1.566	2.9305
Age	0.021	0.003	<0.001	0.011	0.003	<0.001	0.013	0.004	0.0005	0.000	0.002	4.8575	0.000		0.003	4.36	−0.139		0.059	0.091
Current treatment																				
TTFields only																				
TTFields + others	0.101	0.076	0.9255	0.139	0.069	0.2145	0.205	0.087	0.091	0.120	0.058	0.1995	0.039		0.062	2.636	−4.143		1.473	0.0245
Time since diagnosis																				
0–15 months																				
>15 months	0.079	0.076	1.501	−0.028	0.069	3.4065	−0.150	0.088	0.441	−0.002	0.059	4.835	−0.082		0.063	0.9725	2.639		1.492	0.385
Time since diagnosis	0.003	0.002	0.1865	0.003	0.002	0.4365	0.000	0.002	4.7195	0.002	0.001	0.662	0.000		0.001	4.219	0.008		0.032	4.0295
Log(Time since diagnosis)	0.112	0.052	0.1655	0.034	0.048	2.394	−0.033	0.061	2.913	0.035	0.040	1.9485	−0.022		0.044	3.069	0.992		1.030	1.6775
Time on TTFields	−0.0032	0.003	1.371	−0.007	0.003	0.0405	−0.010	0.003	0.019	−0.004	0.002	0.389	−0.005		0.002	0.2655	0.175		0.056	0.009
Log(Time on TTFields)	−0.028	0.040	2.402	−0.086	0.036	0.0795	−0.126	0.046	0.029	−0.045	0.030	0.6905	−0.043		0.032	0.8975	2.109		0.768	0.03
***Progressed***																				
Gender																				
Female (ref.)																				
Male	0.106	0.169	2.654	0.199	0.174	1.2615	0.282	0.165	0.434	−0.058	0.116	3.0945	−0.069		0.111	2.672	−4.395		3.135	0.8045
Age	0.021	0.007	0.009	0.017	0.007	0.0985	0.021	0.007	0.0115	−0.005	0.005	1.2645	−0.001		0.005	4.1515	−0.297		0.129	0.1045
Current treatment																				
TTFields only																				
TTFields + others	−0.087	0.173	3.087	−0.080	0.181	3.292	−0.095	0.172	2.902	0.087	0.122	2.387	0.000		0.114	5	2.879		3.283	1.9025
Time since diagnosis																				
0–15 months																				
>15 months	−0.375	0.156	0.081	−0.553	0.161	0.003	−0.524	0.153	0.003	−0.008	0.110	4.7015	−0.095		0.105	1.837	6.917		2.978	0.101
Time since diagnosis	−0.006	0.003	0.1875	−0.008	0.003	0.0185	−0.007	0.003	0.034	−0.002	0.002	1.6985	−0.002		0.002	1.633	0.169		0.051	0.0045
Log(Time since diagnosis)	−0.239	0.099	0.079	−0.333	0.102	0.0055	−0.315	0.097	0.0055	−0.026	0.070	3.563	−0.037		0.067	2.8815	5.016		1.925	0.046
Time on TTFields	−0.013	0.006	0.155	−0.019	0.006	0.009	−0.016	0.006	0.035	−0.003	0.004	2.2505	0.001		0.004	4.316	0.244		0.115	0.167
Log(Time on TTFields)	−0.223	0.081	0.03	−0.264	0.084	0.0085	−0.217	0.079	0.0305	−0.055	0.058	1.707	−0.053		0.055	1.693	2.623		1.563	0.4665

Entries with light shading denote statistically significant deterioration, while those with dark shading indicate statistically significant improvement. Within the EQ-5D subscales (mobility, self-care, usual activities, pain/discomfort, and anxiety/depression), lower scores indicate less impairment and higher scores more impairment in health-related quality of life (HRQoL). For EQ-VAS, higher values indicate improved self-rated health and lower values indicate worse self-rated health. Quoted p**-**values = raw p-values × 5 to adjust for multiple comparisons.

VAS, visual analogue scale; TTFields, tumor treating fields.

Time since diagnosis did not display significant effects in the non-progressed subgroup. However, patients with progressed disease and longer time since diagnosis reported significantly better self-care (*p* = 0.003), usual activities (*p* = 0.003), and EQ-VAS (*p* = 0.0045).

Longer time on TTFields had a significant positive association with self-care (*p* = 0.0405 and *p* = 0.0085) and usual activities (*p* = 0.019 and *p* = 0.0305) in the non-progressed and progressed subgroups, respectively. A positive association with EQ-VAS (*p* = 0.009) was observed in the non-progressed subgroup. Within the progressed subgroup, longer time using TTFields was also associated with improved mobility (*p* = 0.03).

In the multivariate subgroup analysis, age was observed to maintain its negative association with mobility, self-care, usual activities, and EQ-VAS in both non-progressed and progressed subgroups (**Tables 7** and **8**). Negative associations from using other GBM treatments in addition to TTFields were observed only in the non-progressed subgroup.

**Table 7:** Multivariate regression results for the non-progressed and progressed subgroups with time since diagnosis.

Multivariate regression	Mobility			Self-care			Usual activities			Pain/discomfort			Anxiety/depression			VAS			
	Effect	SE	*p*-value	Effect	SE	*p*-value	Effect	SE	*p*-value	Effect	SE	*p*-value	Effect	SE	*p*-value	Effect	SE	*p*-value
***Non-progressed***																		
Gender																		
Female (ref.)																		
Male	−0.090	0.079	0.5118	−0.040	0.074	1.171	0.085	0.094	0.7234	−0.030	0.063	1.2604	−0.043	0.068	1.0436	−0.295	1.612	1.7096
Age	0.021	0.003	<0.001	0.012	0.003	<0.001	0.014	0.004	<0.001	0.000	0.002	1.9988	0.000	0.003	1.9844	−0.150	0.061	0.0278
Current treatment																		
TTFields only																		
TTFields + others	0.127	0.079	0.218	0.167	0.074	0.0466	0.199	0.093	0.0656	0.139	0.062	0.0512	0.039	0.067	1.1294	−3.796	1.600	0.0354
Time since diagnosis	0.005	0.002	0.0082	0.004	0.002	0.0338	0.001	0.002	1.0682	0.003	0.001	0.1172	0.001	0.002	1.411	−0.002	0.036	1.8988
Gender																		
Female (ref.)																		
Male	−0.091	0.079	0.5064	−0.040	0.074	1.1836	0.086	0.094	0.7212	−0.030	0.063	1.2592	−0.043	0.068	1.053	−0.305	1.612	1.6994
Age	0.021	0.003	<0.001	0.011	0.003	<0.001	0.014	0.004	<0.001	0.000	0.002	1.8524	0.000	0.003	1.937	−0.150	0.061	0.0282
Current treatment																		
TTFields only																		
TTFields + others	0.177	0.085	0.0762	0.183	0.080	0.0428	0.197	0.100	0.1	0.164	0.067	0.0296	0.027	0.073	1.4222	−3.593	1.726	0.0748
Log(Time since diagnosis)	0.165	0.059	0.0106	0.092	0.055	0.191	0.018	0.070	1.5838	0.084	0.047	0.144	−0.009	0.050	1.7116	0.288	1.198	1.6204
***Progressed***																		
Gender																		
Female (ref.)																		
Male	0.064	0.173	1.426	0.121	0.179	0.9986	0.236	0.168	0.3204	−0.098	0.121	0.8362	−0.120	0.114	0.5812	−1.431	3.102	1.2894
Age	0.023	0.007	0.0036	0.017	0.008	0.056	0.021	0.007	0.0072	−0.006	0.005	0.4914	−0.003	0.005	1.0734	−0.272	0.132	0.0778
Current treatment																		
TTFields only																		
TTFields + others	−0.165	0.178	0.709	−0.167	0.186	0.7382	−0.136	0.175	0.8748	0.123	0.127	0.6668	0.009	0.118	1.878	4.662	3.232	0.2984
Time since diagnosis	−0.005	0.003	0.113	−0.007	0.003	0.0158	−0.006	0.003	0.047	−0.002	0.002	0.6616	−0.002	0.002	0.445	0.164	0.050	0.002
Gender																		
Female (ref.)																		
Male	0.085	0.171	1.2402	0.152	0.177	0.7806	0.258	0.166	0.2408	−0.083	0.121	0.9828	−0.106	0.113	0.6928	−2.365	3.105	0.8928
Age	0.023	0.007	0.0038	0.017	0.008	0.0602	0.021	0.007	0.0078	−0.006	0.005	0.5102	−0.003	0.005	1.0894	−0.271	0.133	0.0846
Current treatment																		
TTFields only																		
TTFields + others	−0.218	0.182	0.4656	−0.239	0.190	0.4174	−0.200	0.179	0.5264	0.135	0.130	0.6012	0.006	0.122	1.9194	5.110	3.340	0.252
Log(Time since diagnosis)	−0.222	0.105	0.0694	−0.320	0.109	0.007	−0.271	0.103	0.0162	−0.013	0.075	1.7214	−0.053	0.070	0.9108	4.708	1.967	0.0334

Entries with light shading denote statistically significant deterioration, while those with dark shading indicate statistically significant improvement. Within the EQ-5D subscales (mobility, self-care, usual activities, pain/discomfort, and anxiety/depression), lower scores indicate less impairment and higher scores more impairment in health-related quality of life (HRQoL). For EQ-VAS, higher values indicate improved self-rated health and lower values indicate worse self-rated health. Quoted p-values = raw p-vales × 2 to adjust for multiple comparisons.

VAS, visual analogue scale; TTFields, tumor treating fields.

**Table 8:** Multivariate regression results for the non-progressed and progressed subgroups with time on TTFields.

Multivariate regression	Mobility			Self-care			Usual activities			Pain/discomfort			Anxiety/depression			VAS		
	Effect	SE	*p*-value	Effect	SE	*p*-value	Effect	SE	*p*-value	Effect	SE	*p*-value	Effect	SE	*p*-value	Effect	SE	*p*-value
***Non-progressed***																		
Gender																		
Female (ref.)																		
Male	−0.063	0.083	0.9002	0.021	0.076	1.5748	0.089	0.096	0.7056	−0.019	0.065	1.5478	−0.028	0.070	1.3758	−0.284	1.649	1.7262
Age	0.021	0.003	<0.001	0.010	0.003	0.0006	0.014	0.004	<0.001	0.001	0.002	1.411	0.001	0.003	1.6354	−0.142	0.062	0.043
Current treatment																		
TTFields only																		
TTFields + others	0.062	0.088	0.962	0.048	0.081	1.1042	0.106	0.101	0.5918	0.084	0.068	0.4344	−0.014	0.074	1.695	−1.913	1.735	0.5406
Time on TTFields	−0.001	0.003	1.5832	−0.006	0.003	0.137	−0.008	0.004	0.0846	−0.002	0.003	0.8242	−0.004	0.003	0.3104	0.151	0.065	0.0394
Gender																		
Female (ref.)																		
Male	−0.068	0.083	0.8232	0.016	0.077	1.6668	0.082	0.096	0.7924	−0.025	0.065	1.397	−0.030	0.070	1.3306	−0.238	1.654	1.771
Age	0.021	0.003	<0.001	0.011	0.003	0.0004	0.015	0.004	<0.001	0.0011	0.002	1.3072	0.0009	0.003	1.4432	−0.154	0.062	0.0258
Current treatment																		
TTFields only																		
TTFields + others	0.064	0.091	0.9696	0.046	0.084	1.1766	0.096	0.105	0.719	0.098	0.071	0.3352	−0.005	0.077	1.9014	−1.838	1.795	0.6114
Log(Time on TTFields)	−0.018	0.046	1.4058	−0.074	0.043	0.163	−0.117	0.053	0.0558	−0.018	0.036	1.2316	−0.040	0.039	0.5988	1.998	0.912	0.057
***Progressed***																		
Gender																		
Female (ref.)																		
Male	0.047	0.174	1.5754	0.130	0.181	0.947	0.203	0.169	0.456	−0.103	0.125	0.8248	−0.104	0.117	0.7458	−1.556	3.186	1.2504
Age	0.024	0.008	0.0022	0.020	0.008	0.025	0.025	0.007	0.0014	−0.005	0.005	0.79	−0.004	0.005	0.9358	−0.338	0.137	0.0274
Current treatment																		
TTFields only																		
TTFields + others	−0.215	0.189	0.5096	−0.241	0.197	0.4418	−0.204	0.184	0.5362	0.136	0.137	0.6422	0.038	0.127	1.5252	5.722	3.494	0.203
Time on TTFields	−0.015	0.006	0.0386	−0.021	0.007	0.003	−0.017	0.006	0.0142	−0.002	0.005	1.4742	0.001	0.004	1.67	0.271	0.118	0.044
Gender																		
Female (ref.)																		
Male	0.039	0.174	1.6426	0.118	0.181	1.0298	0.195	0.169	0.496	−0.109	0.125	0.7696	−0.107	0.117	0.7178	−1.351	3.200	1.346
Age	0.025	0.007	0.0014	0.020	0.008	0.0206	0.025	0.007	0.0014	−0.0041	0.005	0.9004	−0.0027	0.005	1.1764	−0.342	0.138	0.0262
Current treatment																		
TTFields only																		
TTFields + others	−0.263	0.189	0.3284	−0.259	0.199	0.386	−0.214	0.186	0.4986	0.119	0.138	0.7782	−0.018	0.128	1.7724	5.304	3.530	0.266
Log(Time on TTFields)	−0.240	0.088	0.0126	−0.274	0.092	0.0058	−0.213	0.086	0.026	−0.035	0.063	1.1624	−0.061	0.060	0.6092	2.540	1.633	0.2396

Entries with light shading denote statistically significant deterioration, while those with dark shading indicate statistically significant improvement. Within the EQ-5D subscales (mobility, self-care, usual activities, pain/discomfort, and anxiety/depression), lower scores indicate less impairment and higher scores more impairment in health-related quality of life (HRQoL). For EQ-VAS, higher values indicate improved self-rated health and lower values indicate worse self-rated health. Quoted p-values = raw p-vales × 2 to adjust for multiple comparisons.

VAS, visual analogue scale; TTFields, tumor treating fields.

Within the non-progressed subgroup, longer time since diagnosis was negatively associated with mobility (*p* = 0.0082) and self-care (*p* = 0.0338). However, a significant positive association between self-care (*p* = 0.007), usual activities (*p* = 0.0162), and EQ-VAS (*p* = 0.002) with longer time since diagnosis was found in the progressed subgroup (**Table 7**).

In multivariate regressions for both the non-progressed and progressed subgroups, longer time on TTFields was observed to have a significant positive association with EQ-VAS (*p* = 0.0394 and *p* = 0.044, respectively) (**Table 8**). In the progressed subgroup, the positive association with longer time on TTFields was larger, more significant, and applied to more dimensions. In particular, patients with progressed disease and longer time on TTFields additionally reported significantly improved mobility (*p* = 0.0126), self-care (*p* = 0.003), and usual activities (*p* = 0.0142).

### Ordinal Regressions for the EQ-5D-5L Dimensions

Univariate and multivariate ordinal regressions were performed to validate the findings of the linear regressions (**Supplementary Tables S1–S6**). Overall, the results were essentially the same in both settings. Disease progression had a significant negative impact on all five dimensions, and age had a significant negative association with mobility, self-care, and usual activities in both progressed and non-progressed subgroups. Additionally, longer time since diagnosis was found to have significant positive associations with self-care and usual activities, and longer time on TTFields had significant positive associations with mobility, self-care, and usual activities. A type of reversal was seen in the non-progressed *vs.* the progressed subgroup, with longer time since diagnosis being negatively associated with mobility in the former and positively with self-care and usual activities in the latter. Longer time on TTFields was seen to have a stronger positive association with mobility, self-care, and usual activities in patients reporting progressed disease as well.

## Discussion

A total of 1,106 responses were included in this analysis, making it the largest real-world sample of patient-reported HRQoL in GBM, to the authors’ knowledge. Our analyses showed an unsurprising negative effect of disease progression on overall patient-reported HRQoL and all of the measured dimensions. However, our analyses additionally uncovered several interesting results. Firstly, longer time since diagnosis and longer time using TTFields were often associated with a positive effect on HRQoL, with the latter having a stronger association. When the patients were divided into the non-progressed and progressed subgroups, time since diagnosis was generally negatively associated with HRQoL in the non-progressed subgroup and positively within the progressed subgroup. Time on TTFields generally exhibited a positive association with HRQoL for both non-progressed and progressed subgroups, with a stronger positive effect size measured in the latter.

Our findings demonstrating the negative effect of age and progression on HRQoL are consistent with those reported in the literature (17, 22). However, the positive effects associated with time since diagnosis and time on TTFields have not been previously reported and may run contrary to the general perception within GBM. This analysis showed a positive relationship between time since diagnosis of >15 months on self-care, usual activities, and EQ-VAS compared to <15 months for patients with GBM currently receiving TTFields. Prior studies of quality of life in long-term cancer survivors have shown that quality of life can return to levels close to those of the general population, which may explain the trend in improved HRQoL for longer time since diagnosis (24).

Time on TTFields was associated with positive effects on mobility, self-care, usual activities, and EQ-VAS in this study. The analysis of the global health status in the EF-14 trial reported no significant increase or decrease from baseline to 12 months for both the TTFields + temozolomide arm and the temozolomide-alone arm (14). However, there was a limited time horizon available in the EF-14 trial, and HRQoL was measured through the validated EORTC QLQ-C30 and brain module (QLQ-BN20) in the EF-14 trial rather than the EQ-5D as in this survey (14).

An important finding of our study was within the subgroup of patients reporting progressed disease. As expected, time since diagnosis was generally negatively associated with HRQoL in patients reporting non-progressed disease. However, patients reporting progressed disease generally had positive associations between time since diagnosis and HRQoL, specifically for self-care, usual activities, and EQ-VAS. Previous studies have shown that progression within GBM is associated with cognitive decline (25) and deterioration of HRQoL over time (22). Although time since diagnosis was associated with a negative effect on HRQoL pre-progression, time on TTFields was associated with a positive effect both pre- and post-progression, with a stronger positive effect in the latter.

Patients were not asked about reasons for continuing TTFields therapy after progression of their disease; however, the above results are not wholly unexpected. The EF-14 trial showed clinical benefit from TTFields in patients after disease progression. In particular, TTFields + chemotherapy was shown to significantly increase the overall survival compared to chemotherapy alone in patients after disease progression in a *post-hoc* analysis of the EF-14 trial (26).

This study provides patients and clinicians insight into the largest known HRQoL survey of long-term GBM survivors. Prior to the publication of these data, HRQoL was largely unknown for this group. Although baseline HRQoL to better understand the evolution of HRQoL over time was unavailable, the positive associations between HRQoL and time on TTFields and time since diagnosis are encouraging and may complement clinical decision-making with patient-reported information.

This study has several limitations. Firstly, the patients included in this survey were currently receiving treatment with TTFields, and the results may not apply to the general GBM population. Furthermore, there may have been a response bias as the response rate was 39.4% and the sample may not be representative of all patients receiving TTFields for GBM in the USA and Europe. For example, the respondents may be patients who have better functional status at the time of the survey, while patients who did not respond to the survey may be older with worst functional status than those who responded. Secondly, in addition to the EQ-5D-5L and EQ-VAS surveys, the patient demographics and clinical information were also self-reported and not able to be validated with medical records. Thirdly, this was a cross-sectional survey evaluating HRQoL at a single point in time. Therefore, the results of this analysis should not necessarily be used to understand HRQoL over time on an individual patient level. Lastly, as this study only included patients actively using TTFields, the effect of time since diagnosis on patients not receiving TTFields is unknown. It is possible that there are other factors, including supportive care (e.g., dexamethasone or anti-epileptic drugs) and time using chemo- and radiotherapy, associated with the improvement in HRQoL over time, which were not measured as part of this survey.

The design of this study was a compromise that tried to maintain ease of completion for patients, compliance with regard to off-label speech and patient privacy, and to collect as much relevant information as possible. The brevity and relative ease of the survey led to a large number of patient responses, but inevitably meant some details remain unknown about each patient’s disease and treatment characteristics.

## Conclusion

This is the largest study to date of real-world cross-sectional HRQoL outcomes reported from patients with GBM receiving treatment with TTFields. Its results show significant negative associations between HRQoL and disease progression, as well as older age. It additionally shows positive associations between HRQoL and longer time since diagnosis and longer time using TTFields therapy, especially in patients with progressed disease. More research is needed to better identify and understand longitudinal effects on patient HRQoL from disease and treatment characteristics, as well as to provide comparisons with patients who are not using TTFields therapy.

## Data Availability Statement

The raw data supporting the conclusions of this article will be made available by the authors, without undue reservation.

## Ethics Statement

The studies involving human participants were reviewed and approved by WCG IRB. The patients/participants provided written informed consent to participate in this study.

## Author Contributions

GC, WF, BW, CP, and C-HT were responsible for the survey and study design. JP, GC, WF, P-YC, and C-HT performed the data analysis. JP, GC, WF, P-YC, and CP contributed to data visualization. JP, GC, WF, and CP wrote the manuscript. All authors contributed to the article and approved the submitted version.

## Funding

This study was funded by Novocure.

## Conflict of Interest

GC and CP are employed by Novocure. WF, BW, and P-YC are paid consultants to Novocure and are employed by Real Chemistry.

The remaining authors declare that the research was conducted in the absence of any commercial or financial relationships that could be construed as a potential conflict of interest.

The authors declare that this study received funding from Novocure. The funder had the following involvement with the study: GC and CP are employed by Novocure.

## Publisher’s Note

All claims expressed in this article are solely those of the authors and do not necessarily represent those of their affiliated organizations, or those of the publisher, the editors and the reviewers. Any product that may be evaluated in this article, or claim that may be made by its manufacturer, is not guaranteed or endorsed by the publisher.
