# Supplementary material for: Health-Related Quality of Life for Patients Receiving Tumor Treating Fields for Glioblastoma
Source: Front Oncol. 2021 Dec 2;11:772261. doi: 10.3389/fonc.2021.772261 (PMC8675863; doi:10.3389/fonc.2021.772261)
Supplement: Supplementary file 1 [file Table_1.docx]

# Supplementary Materials: EQ-5D-5L Ordinal RegressionS

Supplementary Materials: Table 1 Univariate Ordinal Regression Results

*Within the EQ-5D subscales (mobility, self-care, usual activity, pain/discomfort, and anxiety/depression), lower scores indicated less impairment and higher scores more impairment in HRQoL. For EQ-VAS, higher values indicated improved self-rated health and lower values indicated worse self-rated health. Quoted P-values=Raw P-values* $\times$ *6 to adjust for multiple comparisons.*

| **Univariate Regression** | **Mobility** | | | |  | **Self-Care** | | | |  | **Usual Activity** | | | |  | **Pain/Discomfort** | | | |  | **Anxiety Depression** | | | |  |
| --- | --- | --- | --- | --- | --- | --- | --- | --- | --- | --- | --- | --- | --- | --- | --- | --- | --- | --- | --- | --- | --- | --- | --- | --- | --- |
|  | **Adjusted OR** | **95% CI** | | **P-Value** |  | **Adjusted OR** | **95% CI** | | **P-Value** |  | **Adjusted OR** | **95% CI** | | **P-Value** |  | **Adjusted OR** | **95% CI** | | **P-Value** |  | **Adjusted OR** | **95% CI** | | **P-Value** |  |
| Gender |  |  |  |  |  |  |  |  |  |  |  |  |  |  |  |  |  |  |  |  |  |  |  |  |  |
| Female (Ref) |  |  |  |  |  |  |  |  |  |  |  |  |  |  |  |  |  |  |  |  |  |  |  |  |  |
| Male | 0.876 | 0.692 | 1.108 | 1.6194 |  | 1.068 | 0.827 | 1.379 | 3.6948 |  | 1.158 | 0.923 | 1.454 | 1.233 |  | 0.856 | 0.674 | 1.087 | 1.2102 |  | 0.805 | 0.636 | 1.020 | 0.4368 |  |
| Age | 1.041 | 1.031 | 1.052 | <.001 |  | 1.024 | 1.014 | 1.035 | <.001 |  | 1.021 | 1.012 | 1.031 | <.001 |  | 0.997 | 0.988 | 1.006 | 3.078 |  | 0.999 | 0.990 | 1.008 | 5.043 |  |
| Progression Status |  |  |  |  |  |  |  |  |  |  |  |  |  |  |  |  |  |  |  |  |  |  |  |  |  |
| Non-Progressed |  |  |  |  |  |  |  |  |  |  |  |  |  |  |  |  |  |  |  |  |  |  |  |  |  |
| Progressed | 2.680 | 2.076 | 3.459 | <.001 |  | 3.148 | 2.403 | 4.124 | <.001 |  | 2.735 | 2.130 | 3.513 | <.001 |  | 1.819 | 1.406 | 2.353 | <.001 |  | 2.024 | 1.567 | 2.614 | <.001 |  |
| Current Treatment |  |  |  |  |  |  |  |  |  |  |  |  |  |  |  |  |  |  |  |  |  |  |  |  |  |
| TTFields Only |  |  |  |  |  |  |  |  |  |  |  |  |  |  |  |  |  |  |  |  |  |  |  |  |  |
| TTFields + Others | 1.416 | 1.122 | 1.786 | 0.0204 |  | 1.546 | 1.198 | 1.996 | 0.0048 |  | 1.530 | 1.223 | 1.913 | 0.0012 |  | 1.467 | 1.157 | 1.859 | 0.009 |  | 1.259 | 0.999 | 1.587 | 0.3072 |  |
| Time Since Diagnosis |  |  |  |  |  |  |  |  |  |  |  |  |  |  |  |  |  |  |  |  |  |  |  |  |  |
| 0-15 Months |  |  |  |  |  |  |  |  |  |  |  |  |  |  |  |  |  |  |  |  |  |  |  |  |  |
| > 15 Months | 0.892 | 0.712 | 1.118 | 1.923 |  | 0.714 | 0.558 | 0.914 | 0.0444 |  | 0.706 | 0.568 | 0.878 | 0.0108 |  | 0.955 | 0.759 | 1.201 | 4.1598 |  | 0.864 | 0.689 | 1.084 | 1.233 |  |
| Time Since Diagnosis | 0.999 | 0.994 | 1.003 | 3.8988 |  | 0.995 | 0.990 | 1.001 | 0.573 |  | 0.997 | 0.992 | 1.001 | 0.7116 |  | 1.000 | 0.996 | 1.005 | 5.2086 |  | 0.999 | 0.995 | 1.004 | 4.7346 |  |
| Log (Time Since Diagnosis) | 0.946 | 0.814 | 1.098 | 2.7948 |  | 0.824 | 0.698 | 0.972 | 0.1302 |  | 0.839 | 0.726 | 0.970 | 0.1068 |  | 1.008 | 0.866 | 1.173 | 5.532 |  | 0.956 | 0.823 | 1.111 | 3.3612 |  |
| Time-on-TTFields | 0.988 | 0.979 | 0.998 | 0.0804 |  | 0.976 | 0.965 | 0.987 | <.001 |  | 0.983 | 0.974 | 0.991 | <.001 |  | 0.991 | 0.982 | 1.000 | 0.372 |  | 0.993 | 0.984 | 1.002 | 0.8022 |  |
| Log (Time-on-TTFields) | 0.845 | 0.752 | 0.950 | 0.0288 |  | 0.739 | 0.651 | 0.840 | <.001 |  | 0.789 | 0.705 | 0.883 | <.001 |  | 0.899 | 0.799 | 1.012 | 0.4728 |  | 0.905 | 0.806 | 1.017 | 0.564 |  |
| Notes: Statistically significant improvement; Statistically significant decline  Abbreviations: CI = Confidence Interval; OR = Odds Ratio; SE = Standard Error | | | | | | | | | | | | | | | | | | | | | | | | | |

Supplementary Materials: Table 2 Multivariate Ordinal Regression Results with Time-Since-Diagnosis

*Within the EQ-5D subscales (mobility, self-care, usual activity, pain/discomfort, and anxiety/depression), lower scores indicated less impairment and higher scores more impairment in HRQoL. For EQ-VAS, higher values indicated improved self-rated health and lower values indicated worse self-rated health. Quoted P-values=Raw P-values* $\times$ *6 to adjust for multiple comparisons.*

| **Multivariate Regression** | **Mobility** | | | |  | **Self-Care** | | | |  | **Usual Activity** | | | |  | **Pain/Discomfort** | | | |  | **Anxiety Depression** | | | |  |
| --- | --- | --- | --- | --- | --- | --- | --- | --- | --- | --- | --- | --- | --- | --- | --- | --- | --- | --- | --- | --- | --- | --- | --- | --- | --- |
|  | **Adjusted OR** | **95% CI** | | **P-Value** |  | **Adjusted OR** | **95% CI** | | **P-Value** |  | **Adjusted OR** | **95% CI** | | **P-Value** |  | **Adjusted OR** | **95% CI** | | **P-Value** |  | **Adjusted OR** | **95% CI** | | **P-Value** |  |
| Gender |  |  |  |  |  |  |  |  |  |  |  |  |  |  |  |  |  |  |  |  |  |  |  |  |  |
| Female (Ref) |  |  |  |  |  |  |  |  |  |  |  |  |  |  |  |  |  |  |  |  |  |  |  |  |  |
| Male | 0.927 | 0.712 | 1.206 | 1.1432 |  | 1.164 | 0.871 | 1.556 | 0.608 |  | 1.309 | 1.019 | 1.680 | 0.0698 |  | 0.857 | 0.659 | 1.114 | 0.4974 |  | 0.825 | 0.637 | 1.068 | 0.287 |  |
| Age | 1.046 | 1.034 | 1.058 | <.001 |  | 1.025 | 1.013 | 1.038 | <.001 |  | 1.025 | 1.014 | 1.035 | <.001 |  | 0.998 | 0.988 | 1.008 | 1.4046 |  | 0.999 | 0.989 | 1.009 | 1.7062 |  |
| Progression Status |  |  |  |  |  |  |  |  |  |  |  |  |  |  |  |  |  |  |  |  |  |  |  |  |  |
| Non-Progressed |  |  |  |  |  |  |  |  |  |  |  |  |  |  |  |  |  |  |  |  |  |  |  |  |  |
| Progressed | 2.926 | 2.210 | 3.873 | <.001 |  | 3.226 | 2.401 | 4.334 | <.001 |  | 2.913 | 2.219 | 3.825 | <.001 |  | 1.794 | 1.357 | 2.372 | <.001 |  | 2.098 | 1.590 | 2.770 | <.001 |  |
| Current Treatment |  |  |  |  |  |  |  |  |  |  |  |  |  |  |  |  |  |  |  |  |  |  |  |  |  |
| TTFields Only |  |  |  |  |  |  |  |  |  |  |  |  |  |  |  |  |  |  |  |  |  |  |  |  |  |
| TTFields + Others | 1.132 | 0.853 | 1.503 | 0.7782 |  | 1.161 | 0.853 | 1.582 | 0.685 |  | 1.120 | 0.858 | 1.461 | 0.8124 |  | 1.470 | 1.107 | 1.951 | 0.0154 |  | 1.018 | 0.771 | 1.343 | 1.8026 |  |
| Time Since Diagnosis |  |  |  |  |  |  |  |  |  |  |  |  |  |  |  |  |  |  |  |  |  |  |  |  |  |
| 0-15 Months |  |  |  |  |  |  |  |  |  |  |  |  |  |  |  |  |  |  |  |  |  |  |  |  |  |
| > 15 Months | 0.990 | 0.751 | 1.305 | 1.8838 |  | 0.709 | 0.524 | 0.958 | 0.0506 |  | 0.708 | 0.545 | 0.920 | 0.0192 |  | 1.107 | 0.840 | 1.459 | 0.9378 |  | 0.850 | 0.647 | 1.115 | 0.4802 |  |
| Notes: Statistically significant improvement; Statistically significant decline  Abbreviations: CI = Confidence Interval; OR = Odds Ratio; SE = Standard Error | | | | | | | | | | | | | | | | | | | | | | | | | |

Supplementary Materials: Table 3 Multivariate Ordinal Regression Results with Time-on-TTFields

*Within the EQ-5D subscales (mobility, self-care, usual activity, pain/discomfort, and anxiety/depression), lower scores indicated less impairment and higher scores more impairment in HRQoL. For EQ-VAS, higher values indicated improved self-rated health and lower values indicated worse self-rated health. Quoted P-values=Raw P-values* $\times$ *6 to adjust for multiple comparisons.*

| **Multivariate Regression** | **Mobility** | | | |  | **Self-Care** | | | |  | **Usual Activity** | | | |  | **Pain/Discomfort** | | | |  | **Anxiety Depression** | | | |  |
| --- | --- | --- | --- | --- | --- | --- | --- | --- | --- | --- | --- | --- | --- | --- | --- | --- | --- | --- | --- | --- | --- | --- | --- | --- | --- |
|  | **Adjusted OR** | **95% CI** | | **P-Value** |  | **Adjusted OR** | **95% CI** | | **P-Value** |  | **Adjusted OR** | **95% CI** | | **P-Value** |  | **Adjusted OR** | **95% CI** | | **P-Value** |  | **Adjusted OR** | **95% CI** | | **P-Value** |  |
| Gender |  |  |  |  |  |  |  |  |  |  |  |  |  |  |  |  |  |  |  |  |  |  |  |  |  |
| Female (Ref) |  |  |  |  |  |  |  |  |  |  |  |  |  |  |  |  |  |  |  |  |  |  |  |  |  |
| Male | 0.921 | 0.701 | 1.212 | 1.1166 |  | 1.255 | 0.927 | 1.699 | 0.2822 |  | 1.259 | 0.972 | 1.630 | 0.1624 |  | 0.842 | 0.641 | 1.106 | 0.4336 |  | 0.837 | 0.641 | 1.093 | 0.3838 |  |
| Age | 1.046 | 1.033 | 1.058 | <.001 |  | 1.025 | 1.013 | 1.038 | <.001 |  | 1.027 | 1.016 | 1.038 | <.001 |  | 1.0010 | 0.991 | 1.012 | 1.644 |  | 1.000 | 0.990 | 1.010 | 1.9856 |  |
| Progression Status |  |  |  |  |  |  |  |  |  |  |  |  |  |  |  |  |  |  |  |  |  |  |  |  |  |
| Non-Progressed |  |  |  |  |  |  |  |  |  |  |  |  |  |  |  |  |  |  |  |  |  |  |  |  |  |
| Progressed | 3.042 | 2.281 | 4.057 | <.001 |  | 3.299 | 2.435 | 4.470 | <.001 |  | 2.935 | 2.222 | 3.878 | <.001 |  | 1.995 | 1.498 | 2.655 | <.001 |  | 2.192 | 1.650 | 2.912 | <.001 |  |
| Current Treatment |  |  |  |  |  |  |  |  |  |  |  |  |  |  |  |  |  |  |  |  |  |  |  |  |  |
| TTFields Only |  |  |  |  |  |  |  |  |  |  |  |  |  |  |  |  |  |  |  |  |  |  |  |  |  |
| TTFields + Others | 1.042 | 0.778 | 1.395 | 1.5638 |  | 1.027 | 0.745 | 1.417 | 1.7388 |  | 1.114 | 0.847 | 1.465 | 0.8812 |  | 1.368 | 1.022 | 1.832 | 0.0708 |  | 1.021 | 0.768 | 1.358 | 1.7698 |  |
| Time-on-TTFields | 0.992 | 0.982 | 1.003 | 0.35 |  | 0.978 | 0.965 | 0.991 | 0.0018 |  | 0.985 | 0.975 | 0.995 | 0.0068 |  | 0.996 | 0.985 | 1.007 | 0.8886 |  | 0.995 | 0.984 | 1.005 | 0.6146 |  |
| **Multivariate Regression** | **Mobility** | | | |  | **Self-Care** | | | |  | **Usual Activity** | | | |  | **Pain/Discomfort** | | | |  | **Anxiety Depression** | | | |  |
|  | **Adjusted OR** | **95% CI** | | **P-Value** |  | **Adjusted OR** | **95% CI** | | **P-Value** |  | **Adjusted OR** | **95% CI** | | **P-Value** |  | **Adjusted OR** | **95% CI** | | **P-Value** |  | **Adjusted OR** | **95% CI** | | **P-Value** |  |
| Gender |  |  |  |  |  |  |  |  |  |  |  |  |  |  |  |  |  |  |  |  |  |  |  |  |  |
| Female (Ref) |  |  |  |  |  |  |  |  |  |  |  |  |  |  |  |  |  |  |  |  |  |  |  |  |  |
| Male | 0.910 | 0.691 | 1.197 | 0.997 |  | 1.240 | 0.916 | 1.679 | 0.3284 |  | 1.240 | 0.957 | 1.607 | 0.2074 |  | 0.828 | 0.630 | 1.088 | 0.3504 |  | 0.834 | 0.638 | 1.090 | 0.3676 |  |
| Age | 1.047 | 1.034 | 1.059 | <.001 |  | 1.026 | 1.014 | 1.039 | <.001 |  | 1.028 | 1.018 | 1.039 | <.001 |  | 1.0020 | 0.991 | 1.013 | 1.5014 |  | 1.0010 | 0.990 | 1.011 | 1.8204 |  |
| Progression Status |  |  |  |  |  |  |  |  |  |  |  |  |  |  |  |  |  |  |  |  |  |  |  |  |  |
| Non-Progressed |  |  |  |  |  |  |  |  |  |  |  |  |  |  |  |  |  |  |  |  |  |  |  |  |  |
| Progressed | 2.984 | 2.237 | 3.982 | <.001 |  | 3.209 | 2.368 | 4.349 | <.001 |  | 2.873 | 2.174 | 3.797 | <.001 |  | 1.947 | 1.462 | 2.594 | <.001 |  | 2.163 | 1.627 | 2.876 | <.001 |  |
| Current Treatment |  |  |  |  |  |  |  |  |  |  |  |  |  |  |  |  |  |  |  |  |  |  |  |  |  |
| TTFields Only |  |  |  |  |  |  |  |  |  |  |  |  |  |  |  |  |  |  |  |  |  |  |  |  |  |
| TTFields + Others | 1.022 | 0.758 | 1.378 | 1.7774 |  | 1.018 | 0.734 | 1.413 | 1.8292 |  | 1.101 | 0.832 | 1.458 | 0.9992 |  | 1.401 | 1.039 | 1.889 | 0.054 |  | 0.996 | 0.744 | 1.335 | 1.961 |  |
| Log (Time-on-TTFields) | 0.883 | 0.762 | 1.024 | 0.1986 |  | 0.759 | 0.647 | 0.892 | 0.0016 |  | 0.806 | 0.701 | 0.926 | 0.0048 |  | 0.967 | 0.836 | 1.119 | 1.3024 |  | 0.907 | 0.785 | 1.048 | 0.3708 |  |
| Notes: Statistically significant improvement; Statistically significant decline  Abbreviations: CI = Confidence Interval; OR = Odds Ratio; SE = Standard Error | | | | | | | | | | | | | | | | | | | | | | | | | |

Supplementary Materials: Table 4 Univariate Ordinal Regression Results for Non-Progressed and Progressed Subgroups

*Within the EQ-5D subscales (mobility, self-care, usual activity, pain/discomfort, and anxiety/depression), lower scores indicated less impairment and higher scores more impairment in HRQoL. For EQ-VAS, higher values indicated improved self-rated health and lower values indicated worse self-rated health. Quoted P-values=Raw P-values* $\times$ *6 to adjust for multiple comparisons.*

| **Non-Progressed** | | | | | | | | | | | | | | | | | | | | | | | | | |
| --- | --- | --- | --- | --- | --- | --- | --- | --- | --- | --- | --- | --- | --- | --- | --- | --- | --- | --- | --- | --- | --- | --- | --- | --- | --- |
| **Univariate Regression** | **Mobility** | | | |  | **Self-Care** | | | |  | **Usual Activity** | | | |  | **Pain/Discomfort** | | | |  | **Anxiety Depression** | | | |  |
|  | **Adjusted OR** | **95% CI** | | **P-Value** |  | **Adjusted OR** | **95% CI** | | **P-Value** |  | **Adjusted OR** | **95% CI** | | **P-Value** |  | **Adjusted OR** | **95% CI** | | **P-Value** |  | **Adjusted OR** | **95% CI** | | **P-Value** |  |
| Gender |  |  |  |  |  |  |  |  |  |  |  |  |  |  |  |  |  |  |  |  |  |  |  |  |  |
| Female (Ref) |  |  |  |  |  |  |  |  |  |  |  |  |  |  |  |  |  |  |  |  |  |  |  |  |  |
| Male | 0.841 | 0.620 | 1.142 | 1.342 |  | 1.128 | 0.792 | 1.607 | 2.514 |  | 1.264 | 0.946 | 1.690 | 0.5665 |  | 0.963 | 0.708 | 1.311 | 4.0635 |  | 0.884 | 0.654 | 1.196 | 2.1235 |  |
| Age | 1.050 | 1.036 | 1.064 | <.001 |  | 1.027 | 1.012 | 1.042 | 0.0015 |  | 1.022 | 1.011 | 1.034 | 0.0005 |  | 1.001 | 0.989 | 1.012 | 4.5435 |  | 1.002 | 0.991 | 1.014 | 3.511 |  |
| Current Treatment |  |  |  |  |  |  |  |  |  |  |  |  |  |  |  |  |  |  |  |  |  |  |  |  |  |
| TTFields Only |  |  |  |  |  |  |  |  |  |  |  |  |  |  |  |  |  |  |  |  |  |  |  |  |  |
| TTFields + Others | 1.320 | 0.983 | 1.774 | 0.326 |  | 1.698 | 1.202 | 2.397 | 0.013 |  | 1.508 | 1.143 | 1.992 | 0.0185 |  | 1.420 | 1.056 | 1.911 | 0.1015 |  | 1.131 | 0.848 | 1.509 | 2.015 |  |
| Time Since Diagnosis |  |  |  |  |  |  |  |  |  |  |  |  |  |  |  |  |  |  |  |  |  |  |  |  |  |
| 0-15 Months |  |  |  |  |  |  |  |  |  |  |  |  |  |  |  |  |  |  |  |  |  |  |  |  |  |
| > 15 Months | 1.187 | 0.884 | 1.594 | 1.2665 |  | 0.900 | 0.641 | 1.263 | 2.709 |  | 0.787 | 0.596 | 1.039 | 0.4555 |  | 0.937 | 0.698 | 1.259 | 3.3345 |  | 0.842 | 0.630 | 1.125 | 1.225 |  |
| Time Since Diagnosis | 1.006 | 1.000 | 1.012 | 0.2305 |  | 1.004 | 0.997 | 1.010 | 1.513 |  | 1.000 | 0.994 | 1.006 | 4.9 |  | 1.003 | 0.997 | 1.010 | 1.3475 |  | 1.002 | 0.995 | 1.008 | 3.138 |  |
| Log (Time Since Diagnosis) | 1.248 | 1.020 | 1.527 | 0.1575 |  | 1.023 | 0.812 | 1.291 | 4.226 |  | 0.948 | 0.783 | 1.148 | 2.9265 |  | 1.056 | 0.862 | 1.293 | 2.993 |  | 0.973 | 0.797 | 1.188 | 3.942 |  |
| Time-on-TTFields | 0.9950 | 0.984 | 1.007 | 1.954 |  | 0.981 | 0.967 | 0.996 | 0.066 |  | 0.985 | 0.974 | 0.996 | 0.0345 |  | 0.989 | 0.977 | 1.000 | 0.299 |  | 0.990 | 0.978 | 1.001 | 0.3565 |  |
| Log (Time-on-TTFields) | 0.984 | 0.844 | 1.149 | 4.2075 |  | 0.805 | 0.674 | 0.960 | 0.079 |  | 0.824 | 0.712 | 0.953 | 0.045 |  | 0.891 | 0.764 | 1.039 | 0.7055 |  | 0.917 | 0.789 | 1.066 | 1.306 |  |
| **Progressed** | | | | | | | | | | | | | | | | | | | | | | | | | |
| **Univariate Regression** | **Mobility** | | | |  | **Self-Care** | | | |  | **Usual Activity** | | | |  | **Pain/Discomfort** | | | |  | **Anxiety Depression** | | | |  |
|  | **Adjusted OR** | **95% CI** | | **P-Value** |  | **Adjusted OR** | **95% CI** | | **P-Value** |  | **Adjusted OR** | **95% CI** | | **P-Value** |  | **Adjusted OR** | **95% CI** | | **P-Value** |  | **Adjusted OR** | **95% CI** | | **P-Value** |  |
| Gender |  |  |  |  |  |  |  |  |  |  |  |  |  |  |  |  |  |  |  |  |  |  |  |  |  |
| Female (Ref) |  |  |  |  |  |  |  |  |  |  |  |  |  |  |  |  |  |  |  |  |  |  |  |  |  |
| Male | 1.197 | 0.769 | 1.861 | 2.127 |  | 1.287 | 0.817 | 2.026 | 1.385 |  | 1.459 | 0.945 | 2.252 | 0.4425 |  | 0.748 | 0.477 | 1.172 | 1.0225 |  | 0.770 | 0.491 | 1.208 | 1.278 |  |
| Age | 1.032 | 1.013 | 1.051 | 0.0055 |  | 1.023 | 1.004 | 1.043 | 0.1015 |  | 1.026 | 1.008 | 1.045 | 0.024 |  | 0.994 | 0.975 | 1.012 | 2.488 |  | 0.997 | 0.979 | 1.015 | 3.719 |  |
| Current Treatment |  |  |  |  |  |  |  |  |  |  |  |  |  |  |  |  |  |  |  |  |  |  |  |  |  |
| TTFields Only |  |  |  |  |  |  |  |  |  |  |  |  |  |  |  |  |  |  |  |  |  |  |  |  |  |
| TTFields + Others | 0.929 | 0.590 | 1.464 | 3.7605 |  | 0.912 | 0.573 | 1.453 | 3.495 |  | 0.901 | 0.573 | 1.415 | 3.25 |  | 1.229 | 0.765 | 1.976 | 1.9685 |  | 0.953 | 0.598 | 1.519 | 4.194 |  |
| Time Since Diagnosis |  |  |  |  |  |  |  |  |  |  |  |  |  |  |  |  |  |  |  |  |  |  |  |  |  |
| 0-15 Months |  |  |  |  |  |  |  |  |  |  |  |  |  |  |  |  |  |  |  |  |  |  |  |  |  |
| > 15 Months | 0.603 | 0.398 | 0.914 | 0.086 |  | 0.462 | 0.301 | 0.710 | 0.002 |  | 0.493 | 0.326 | 0.746 | 0.004 |  | 1.044 | 0.683 | 1.594 | 4.2175 |  | 0.877 | 0.575 | 1.337 | 2.7095 |  |
| Time Since Diagnosis | 0.991 | 0.983 | 1.000 | 0.215 |  | 0.982 | 0.971 | 0.994 | 0.0145 |  | 0.990 | 0.983 | 0.998 | 0.06 |  | 0.996 | 0.988 | 1.003 | 1.389 |  | 0.996 | 0.989 | 1.004 | 1.7175 |  |
| Log (Time Since Diagnosis) | 0.719 | 0.551 | 0.940 | 0.0785 |  | 0.614 | 0.462 | 0.816 | 0.004 |  | 0.664 | 0.510 | 0.863 | 0.011 |  | 0.957 | 0.731 | 1.254 | 3.7595 |  | 0.948 | 0.725 | 1.240 | 3.481 |  |
| Time-on-TTFields | 0.983 | 0.967 | 1.000 | 0.2285 |  | 0.972 | 0.954 | 0.990 | 0.012 |  | 0.979 | 0.964 | 0.995 | 0.05 |  | 0.995 | 0.979 | 1.011 | 2.786 |  | 1.002 | 0.986 | 1.018 | 4.2545 |  |
| Log (Time-on-TTFields) | 0.739 | 0.594 | 0.918 | 0.032 |  | 0.688 | 0.549 | 0.862 | 0.006 |  | 0.752 | 0.607 | 0.931 | 0.045 |  | 0.917 | 0.736 | 1.144 | 2.215 |  | 0.894 | 0.717 | 1.114 | 1.59 |  |
| Notes: Statistically significant improvement; Statistically significant decline  Abbreviations: CI = Confidence Interval; OR = Odds Ratio; SE = Standard Error | | | | | | | | | | | | | | | | | | | | | | | | | |

Supplementary Materials: Table 5 Multivariate Ordinal Regression Results for Non-Progressed and Progressed Subgroups with Time-Since-Diagnosis

*Within the EQ-5D subscales (mobility, self-care, usual activity, pain/discomfort, and anxiety/depression), lower scores indicated less impairment and higher scores more impairment in HRQoL. For EQ-VAS, higher values indicated improved self-rated health and lower values indicated worse self-rated health. Quoted P-values=Raw P-values* $\times$ *6 to adjust for multiple comparisons.*

| **Non-Progressed** | | | | | | | | | | | | | | | | | | | | | | | | | |
| --- | --- | --- | --- | --- | --- | --- | --- | --- | --- | --- | --- | --- | --- | --- | --- | --- | --- | --- | --- | --- | --- | --- | --- | --- | --- |
| **Multivariate Regression** | **Mobility** | | | |  | **Self-Care** | | | |  | **Usual Activity** | | | |  | **Pain/Discomfort** | | | |  | **Anxiety Depression** | | | |  |
|  | **Adjusted OR** | **95% CI** | | **P-Value** |  | **Adjusted OR** | **95% CI** | | **P-Value** |  | **Adjusted OR** | **95% CI** | | **P-Value** |  | **Adjusted OR** | **95% CI** | | **P-Value** |  | **Adjusted OR** | **95% CI** | | **P-Value** |  |
| Gender |  |  |  |  |  |  |  |  |  |  |  |  |  |  |  |  |  |  |  |  |  |  |  |  |  |
| Female (Ref) |  |  |  |  |  |  |  |  |  |  |  |  |  |  |  |  |  |  |  |  |  |  |  |  |  |
| Male | 0.826 | 0.597 | 1.143 | 0.497 |  | 1.056 | 0.730 | 1.527 | 1.5424 |  | 1.226 | 0.907 | 1.656 | 0.3702 |  | 0.937 | 0.681 | 1.290 | 1.3802 |  | 0.871 | 0.638 | 1.189 | 0.7692 |  |
| Age | 1.053 | 1.038 | 1.068 | <.001 |  | 1.028 | 1.013 | 1.044 | 0.0006 |  | 1.024 | 1.012 | 1.036 | <.001 |  | 1.001 | 0.989 | 1.013 | 1.8374 |  | 1.002 | 0.990 | 1.014 | 1.579 |  |
| Current Treatment |  |  |  |  |  |  |  |  |  |  |  |  |  |  |  |  |  |  |  |  |  |  |  |  |  |
| TTFields Only |  |  |  |  |  |  |  |  |  |  |  |  |  |  |  |  |  |  |  |  |  |  |  |  |  |
| TTFields + Others | 1.470 | 1.061 | 2.035 | 0.0408 |  | 1.815 | 1.255 | 2.623 | 0.003 |  | 1.531 | 1.134 | 2.066 | 0.0108 |  | 1.521 | 1.105 | 2.093 | 0.0202 |  | 1.157 | 0.848 | 1.578 | 0.7174 |  |
| Time Since Diagnosis | 1.012 | 1.005 | 1.019 | 0.0026 |  | 1.008 | 1.000 | 1.015 | 0.0932 |  | 1.003 | 0.996 | 1.009 | 0.8808 |  | 1.006 | 0.999 | 1.013 | 0.202 |  | 1.002 | 0.996 | 1.010 | 0.9726 |  |
| **Multivariate Regression** | **Mobility** | | | |  | **Self-Care** | | | |  | **Usual Activity** | | | |  | **Pain/Discomfort** | | | |  | **Anxiety Depression** | | | |  |
|  | **Adjusted OR** | **95% CI** | | **P-Value** |  | **Adjusted OR** | **95% CI** | | **P-Value** |  | **Adjusted OR** | **95% CI** | | **P-Value** |  | **Adjusted OR** | **95% CI** | | **P-Value** |  | **Adjusted OR** | **95% CI** | | **P-Value** |  |
| Gender |  |  |  |  |  |  |  |  |  |  |  |  |  |  |  |  |  |  |  |  |  |  |  |  |  |
| Female (Ref) |  |  |  |  |  |  |  |  |  |  |  |  |  |  |  |  |  |  |  |  |  |  |  |  |  |
| Male | 0.824 | 0.595 | 1.139 | 0.482 |  | 1.060 | 0.733 | 1.533 | 1.514 |  | 1.227 | 0.908 | 1.657 | 0.3666 |  | 0.940 | 0.683 | 1.295 | 1.4116 |  | 0.872 | 0.639 | 1.191 | 0.7792 |  |
| Age | 1.052 | 1.037 | 1.067 | <.001 |  | 1.028 | 1.012 | 1.043 | 0.0008 |  | 1.024 | 1.012 | 1.036 | <.001 |  | 1.000 | 0.988 | 1.012 | 1.9672 |  | 1.001 | 0.990 | 1.013 | 1.6214 |  |
| Current Treatment |  |  |  |  |  |  |  |  |  |  |  |  |  |  |  |  |  |  |  |  |  |  |  |  |  |
| TTFields Only |  |  |  |  |  |  |  |  |  |  |  |  |  |  |  |  |  |  |  |  |  |  |  |  |  |
| TTFields + Others | 1.680 | 1.181 | 2.392 | 0.0078 |  | 1.925 | 1.292 | 2.867 | 0.0026 |  | 1.533 | 1.110 | 2.118 | 0.0192 |  | 1.626 | 1.151 | 2.298 | 0.0118 |  | 1.138 | 0.813 | 1.592 | 0.904 |  |
| Log (Time Since Diagnosis) | 1.501 | 1.175 | 1.916 | 0.0022 |  | 1.248 | 0.953 | 1.635 | 0.2144 |  | 1.047 | 0.837 | 1.310 | 1.3756 |  | 1.223 | 0.966 | 1.550 | 0.1898 |  | 1.018 | 0.807 | 1.285 | 1.7584 |  |
| Notes: Statistically significant improvement; Statistically significant decline  Abbreviations: CI = Confidence Interval; OR = Odds Ratio; SE = Standard Error | | | | | | | | | | | | | | | | | | | | | | | | | |
| **Progressed** | | | | | | | | | | | | | | | | | | | | | | | | | |
| **Multivariate Regression** | **Mobility** | | | |  | **Self-Care** | | | |  | **Usual Activity** | | | |  | **Pain/Discomfort** | | | |  | **Anxiety Depression** | | | |  |
|  | **Adjusted OR** | **95% CI** | | **P-Value** |  | **Adjusted OR** | **95% CI** | | **P-Value** |  | **Adjusted OR** | **95% CI** | | **P-Value** |  | **Adjusted OR** | **95% CI** | | **P-Value** |  | **Adjusted OR** | **95% CI** | | **P-Value** |  |
| Gender |  |  |  |  |  |  |  |  |  |  |  |  |  |  |  |  |  |  |  |  |  |  |  |  |  |
| Female (Ref) |  |  |  |  |  |  |  |  |  |  |  |  |  |  |  |  |  |  |  |  |  |  |  |  |  |
| Male | 1.092 | 0.687 | 1.734 | 1.4212 |  | 1.181 | 0.733 | 1.903 | 0.9884 |  | 1.386 | 0.880 | 2.182 | 0.3174 |  | 0.683 | 0.427 | 1.093 | 0.2244 |  | 0.694 | 0.434 | 1.110 | 0.255 |  |
| Age | 1.035 | 1.014 | 1.056 | 0.0022 |  | 1.023 | 1.002 | 1.045 | 0.0684 |  | 1.027 | 1.007 | 1.048 | 0.0146 |  | 0.992 | 0.972 | 1.012 | 0.878 |  | 0.992 | 0.972 | 1.012 | 0.8406 |  |
| Current Treatment |  |  |  |  |  |  |  |  |  |  |  |  |  |  |  |  |  |  |  |  |  |  |  |  |  |
| TTFields Only |  |  |  |  |  |  |  |  |  |  |  |  |  |  |  |  |  |  |  |  |  |  |  |  |  |
| TTFields + Others | 0.814 | 0.505 | 1.314 | 0.8006 |  | 0.749 | 0.454 | 1.239 | 0.521 |  | 0.806 | 0.502 | 1.293 | 0.7426 |  | 1.295 | 0.787 | 2.130 | 0.6172 |  | 0.947 | 0.581 | 1.543 | 1.6534 |  |
| Time Since Diagnosis | 0.991 | 0.983 | 1.000 | 0.1062 |  | 0.981 | 0.968 | 0.994 | 0.0074 |  | 0.991 | 0.984 | 0.999 | 0.0636 |  | 0.995 | 0.988 | 1.003 | 0.4586 |  | 0.995 | 0.987 | 1.002 | 0.324 |  |
| **Multivariate Regression** | **Mobility** | | | |  | **Self-Care** | | | |  | **Usual Activity** | | | |  | **Pain/Discomfort** | | | |  | **Anxiety Depression** | | | |  |
|  | **Adjusted OR** | **95% CI** | | **P-Value** |  | **Adjusted OR** | **95% CI** | | **P-Value** |  | **Adjusted OR** | **95% CI** | | **P-Value** |  | **Adjusted OR** | **95% CI** | | **P-Value** |  | **Adjusted OR** | **95% CI** | | **P-Value** |  |
| Gender |  |  |  |  |  |  |  |  |  |  |  |  |  |  |  |  |  |  |  |  |  |  |  |  |  |
| Female (Ref) |  |  |  |  |  |  |  |  |  |  |  |  |  |  |  |  |  |  |  |  |  |  |  |  |  |
| Male | 1.124 | 0.709 | 1.780 | 1.238 |  | 1.227 | 0.763 | 1.974 | 0.796 |  | 1.420 | 0.905 | 2.228 | 0.2552 |  | 0.707 | 0.444 | 1.127 | 0.2902 |  | 0.716 | 0.450 | 1.141 | 0.3204 |  |
| Age | 1.034 | 1.014 | 1.056 | 0.0024 |  | 1.023 | 1.002 | 1.045 | 0.0682 |  | 1.028 | 1.008 | 1.048 | 0.0134 |  | 0.992 | 0.972 | 1.013 | 0.91 |  | 0.992 | 0.972 | 1.012 | 0.8622 |  |
| Current Treatment |  |  |  |  |  |  |  |  |  |  |  |  |  |  |  |  |  |  |  |  |  |  |  |  |  |
| TTFields Only |  |  |  |  |  |  |  |  |  |  |  |  |  |  |  |  |  |  |  |  |  |  |  |  |  |
| TTFields + Others | 0.755 | 0.462 | 1.233 | 0.5226 |  | 0.697 | 0.418 | 1.162 | 0.3328 |  | 0.739 | 0.455 | 1.199 | 0.4416 |  | 1.336 | 0.804 | 2.220 | 0.5286 |  | 0.937 | 0.568 | 1.546 | 1.5992 |  |
| Log (Time Since Diagnosis) | 0.725 | 0.543 | 0.968 | 0.0582 |  | 0.593 | 0.434 | 0.809 | 0.002 |  | 0.696 | 0.526 | 0.922 | 0.0232 |  | 0.976 | 0.732 | 1.302 | 1.7396 |  | 0.880 | 0.659 | 1.176 | 0.7742 |  |
| Notes: Statistically significant improvement; Statistically significant decline  Abbreviations: CI = Confidence Interval; OR = Odds Ratio; SE = Standard Error | | | | | | | | | | | | | | | | | | | | | | | | | |

***Supplementary Materials: Table 6 Multivariate Ordinal Regression Results for Non-Progressed and Progressed Subgroups with Time-on-TTFields***

*Within the EQ-5D subscales (mobility, self-care, usual activity, pain/discomfort, and anxiety/depression), lower scores indicated less impairment and higher scores more impairment in HRQoL. For EQ-VAS, higher values indicated improved self-rated health and lower values indicated worse self-rated health. Quoted P-values=Raw P-values* $\times$ *6 to adjust for multiple comparisons.*

| **Non-Progressed** | | | | | | | | | | | | | | | | | | | | | | | | | |
| --- | --- | --- | --- | --- | --- | --- | --- | --- | --- | --- | --- | --- | --- | --- | --- | --- | --- | --- | --- | --- | --- | --- | --- | --- | --- |
| **Multivariate Regression** | **Mobility** | | | |  | **Self-Care** | | | |  | **Usual Activity** | | | |  | **Pain/Discomfort** | | | |  | **Anxiety Depression** | | | |  |
|  | **Adjusted OR** | **95% CI** | | **P-Value** |  | **Adjusted OR** | **95% CI** | | **P-Value** |  | **Adjusted OR** | **95% CI** | | **P-Value** |  | **Adjusted OR** | **95% CI** | | **P-Value** |  | **Adjusted OR** | **95% CI** | | **P-Value** |  |
| Gender |  |  |  |  |  |  |  |  |  |  |  |  |  |  |  |  |  |  |  |  |  |  |  |  |  |
| Female (Ref) |  |  |  |  |  |  |  |  |  |  |  |  |  |  |  |  |  |  |  |  |  |  |  |  |  |
| Male | 0.852 | 0.607 | 1.195 | 0.7064 |  | 1.251 | 0.848 | 1.846 | 0.517 |  | 1.206 | 0.883 | 1.649 | 0.4786 |  | 0.951 | 0.682 | 1.326 | 1.532 |  | 0.895 | 0.647 | 1.237 | 1.0038 |  |
| Age | 1.051 | 1.036 | 1.066 | <.001 |  | 1.026 | 1.010 | 1.042 | 0.002 |  | 1.026 | 1.014 | 1.038 | <.001 |  | 1.003 | 0.991 | 1.016 | 1.1822 |  | 1.003 | 0.991 | 1.016 | 1.1584 |  |
| Current Treatment |  |  |  |  |  |  |  |  |  |  |  |  |  |  |  |  |  |  |  |  |  |  |  |  |  |
| TTFields Only |  |  |  |  |  |  |  |  |  |  |  |  |  |  |  |  |  |  |  |  |  |  |  |  |  |
| TTFields + Others | 1.314 | 0.916 | 1.885 | 0.2764 |  | 1.376 | 0.912 | 2.077 | 0.257 |  | 1.343 | 0.965 | 1.868 | 0.1604 |  | 1.341 | 0.941 | 1.910 | 0.2084 |  | 1.025 | 0.727 | 1.445 | 1.7792 |  |
| Time-on-TTFields | 1.003 | 0.989 | 1.017 | 1.3134 |  | 0.989 | 0.973 | 1.006 | 0.4254 |  | 0.989 | 0.977 | 1.002 | 0.2078 |  | 0.995 | 0.981 | 1.008 | 0.8544 |  | 0.992 | 0.979 | 1.005 | 0.4626 |  |
| **Multivariate Regression** | **Mobility** | | | |  | **Self-Care** | | | |  | **Usual Activity** | | | |  | **Pain/Discomfort** | | | |  | **Anxiety Depression** | | | |  |
|  | **Adjusted OR** | **95% CI** | | **P-Value** |  | **Adjusted OR** | **95% CI** | | **P-Value** |  | **Adjusted OR** | **95% CI** | | **P-Value** |  | **Adjusted OR** | **95% CI** | | **P-Value** |  | **Adjusted OR** | **95% CI** | | **P-Value** |  |
| Gender |  |  |  |  |  |  |  |  |  |  |  |  |  |  |  |  |  |  |  |  |  |  |  |  |  |
| Female (Ref) |  |  |  |  |  |  |  |  |  |  |  |  |  |  |  |  |  |  |  |  |  |  |  |  |  |
| Male | 0.839 | 0.598 | 1.178 | 0.6214 |  | 1.235 | 0.836 | 1.824 | 0.5768 |  | 1.189 | 0.869 | 1.627 | 0.5588 |  | 0.932 | 0.667 | 1.301 | 1.3548 |  | 0.889 | 0.642 | 1.231 | 0.9578 |  |
| Age | 1.051 | 1.036 | 1.066 | <.001 |  | 1.027 | 1.011 | 1.042 | 0.0016 |  | 1.027 | 1.015 | 1.039 | <.001 |  | 1.0040 | 0.991 | 1.017 | 1.0972 |  | 1.0040 | 0.992 | 1.016 | 1.0268 |  |
| Current Treatment |  |  |  |  |  |  |  |  |  |  |  |  |  |  |  |  |  |  |  |  |  |  |  |  |  |
| TTFields Only |  |  |  |  |  |  |  |  |  |  |  |  |  |  |  |  |  |  |  |  |  |  |  |  |  |
| TTFields + Others | 1.347 | 0.928 | 1.956 | 0.2354 |  | 1.387 | 0.910 | 2.115 | 0.2558 |  | 1.333 | 0.948 | 1.874 | 0.196 |  | 1.417 | 0.983 | 2.041 | 0.1232 |  | 1.068 | 0.748 | 1.524 | 1.4352 |  |
| Log (Time-on-TTFields) | 1.044 | 0.863 | 1.262 | 1.3148 |  | 0.876 | 0.709 | 1.082 | 0.4398 |  | 0.849 | 0.714 | 1.010 | 0.128 |  | 0.973 | 0.810 | 1.169 | 1.5436 |  | 0.941 | 0.786 | 1.126 | 1.0122 |  |
| Notes: Statistically significant improvement; Statistically significant decline  Abbreviations: CI = Confidence Interval; OR = Odds Ratio; SE = Standard Error | | | | | | | | | | | | | | | | | | | | | | | | | |
| **Progressed** | | | | | | | | | | | | | | | | | | | | | | | | | |
| **Multivariate Regression** | **Mobility** | | | |  | **Self-Care** | | | |  | **Usual Activity** | | | |  | **Pain/Discomfort** | | | |  | **Anxiety Depression** | | | |  |
|  | **Adjusted OR** | **95% CI** | | **P-Value** |  | **Adjusted OR** | **95% CI** | | **P-Value** |  | **Adjusted OR** | **95% CI** | | **P-Value** |  | **Adjusted OR** | **95% CI** | | **P-Value** |  | **Adjusted OR** | **95% CI** | | **P-Value** |  |
| Gender |  |  |  |  |  |  |  |  |  |  |  |  |  |  |  |  |  |  |  |  |  |  |  |  |  |
| Female (Ref) |  |  |  |  |  |  |  |  |  |  |  |  |  |  |  |  |  |  |  |  |  |  |  |  |  |
| Male | 1.052 | 0.657 | 1.684 | 1.6662 |  | 1.163 | 0.714 | 1.895 | 1.0866 |  | 1.309 | 0.824 | 2.079 | 0.508 |  | 0.678 | 0.420 | 1.095 | 0.224 |  | 0.730 | 0.453 | 1.178 | 0.3956 |  |
| Age | 1.037 | 1.016 | 1.059 | 0.0012 |  | 1.027 | 1.006 | 1.050 | 0.0276 |  | 1.034 | 1.013 | 1.055 | 0.0028 |  | 0.996 | 0.975 | 1.016 | 1.341 |  | 0.990 | 0.970 | 1.011 | 0.7252 |  |
| Current Treatment |  |  |  |  |  |  |  |  |  |  |  |  |  |  |  |  |  |  |  |  |  |  |  |  |  |
| TTFields Only |  |  |  |  |  |  |  |  |  |  |  |  |  |  |  |  |  |  |  |  |  |  |  |  |  |
| TTFields + Others | 0.739 | 0.443 | 1.231 | 0.4894 |  | 0.675 | 0.396 | 1.149 | 0.295 |  | 0.729 | 0.440 | 1.207 | 0.439 |  | 1.366 | 0.805 | 2.318 | 0.4952 |  | 0.996 | 0.592 | 1.676 | 1.9752 |  |
| Time-on-TTFields | 0.979 | 0.962 | 0.997 | 0.0458 |  | 0.966 | 0.946 | 0.986 | 0.002 |  | 0.977 | 0.960 | 0.994 | 0.017 |  | 0.998 | 0.981 | 1.016 | 1.691 |  | 1.000 | 0.982 | 1.018 | 1.9822 |  |
| **Multivariate Regression** | **Mobility** | | | |  | **Self-Care** | | | |  | **Usual Activity** | | | |  | **Pain/Discomfort** | | | |  | **Anxiety Depression** | | | |  |
|  | **Adjusted OR** | **95% CI** | | **P-Value** |  | **Adjusted OR** | **95% CI** | | **P-Value** |  | **Adjusted OR** | **95% CI** | | **P-Value** |  | **Adjusted OR** | **95% CI** | | **P-Value** |  | **Adjusted OR** | **95% CI** | | **P-Value** |  |
| Gender |  |  |  |  |  |  |  |  |  |  |  |  |  |  |  |  |  |  |  |  |  |  |  |  |  |
| Female (Ref) |  |  |  |  |  |  |  |  |  |  |  |  |  |  |  |  |  |  |  |  |  |  |  |  |  |
| Male | 1.040 | 0.649 | 1.667 | 1.7424 |  | 1.157 | 0.709 | 1.888 | 1.1164 |  | 1.288 | 0.810 | 2.047 | 0.5686 |  | 0.667 | 0.413 | 1.077 | 0.195 |  | 0.719 | 0.445 | 1.161 | 0.3542 |  |
| Age | 1.039 | 1.017 | 1.061 | 0.0008 |  | 1.028 | 1.006 | 1.051 | 0.0232 |  | 1.034 | 1.014 | 1.056 | 0.0024 |  | 0.9970 | 0.976 | 1.018 | 1.512 |  | 0.9920 | 0.972 | 1.013 | 0.903 |  |
| Current Treatment |  |  |  |  |  |  |  |  |  |  |  |  |  |  |  |  |  |  |  |  |  |  |  |  |  |
| TTFields Only |  |  |  |  |  |  |  |  |  |  |  |  |  |  |  |  |  |  |  |  |  |  |  |  |  |
| TTFields + Others | 0.686 | 0.409 | 1.151 | 0.3074 |  | 0.660 | 0.386 | 1.128 | 0.2572 |  | 0.722 | 0.434 | 1.201 | 0.4182 |  | 1.325 | 0.778 | 2.258 | 0.6 |  | 0.869 | 0.514 | 1.470 | 1.2012 |  |
| Log (Time-on-TTFields) | 0.708 | 0.555 | 0.901 | 0.0102 |  | 0.647 | 0.503 | 0.833 | 0.0014 |  | 0.745 | 0.588 | 0.944 | 0.0296 |  | 0.957 | 0.751 | 1.218 | 1.439 |  | 0.853 | 0.668 | 1.090 | 0.4064 |  |
| Notes: Statistically significant improvement; Statistically significant decline  Abbreviations: CI = Confidence Interval; OR = Odds Ratio; SE = Standard Error | | | | | | | | | | | | | | | | | | | | | | | | | |
